# H3K18 lactylation in cancer-associated fibroblasts drives malignant pleural effusion progression via TNFR2^+^ T_reg_ recruitment

**DOI:** 10.1038/s12276-025-01557-3

**Published:** 2025-11-28

**Authors:** Linlin Ye, Xuan Xiang, Zihao Wang, Siyu Zhang, Qianqian Xue, Xiaoshan Wei, Yao Liu, Haolei Wang, Jiaqi Ai, Bohan Yang, Long Chen, Yiran Niu, Wenbei Peng, Qiong Zhou

**Affiliations:** 1 Department of Respiratory and Critical Care Medicine, Union Hospital, Tongji Medical College, Huazhong University of Science and Technology, Wuhan, China; 2 Department of Respiratory and Critical Care Medicine, National Clinical Research Center of Respiratory Disease, Key Laboratory of Pulmonary Diseases of Health Ministry, Tongji Hospital, Tongji Medical College, Huazhong University of Science and Technology, Wuhan, China; 3 Department of Respiratory and Critical Care Medicine, The First Affiliated Hospital of Guangxi Medical University, Nanning, China; 4 Department of Respiratory and Critical Care Medicine, The Central Hospital of Wuhan, Tongji Medical College, Huazhong University of Science and Technology, Wuhan, China

**Keywords:** Chemokines, Gene regulation

## Abstract

Tumor necrosis factor receptor 2-positive regulatory T (TNFR2^+^ T_reg_) cells, the most suppressive subset of T_reg_ cells, are enriched in malignant pleural effusion (MPE), contributing to disease progression. However, the underlying mechanisms responsible for their accumulation remain unclear. Here we demonstrate that the C–X–C motif chemokine ligand 16 (CXCL16)/C–X–C chemokine receptor type 6 (CXCR6) axis plays a critical role in recruiting TNFR2^+^ T_reg_ cells to MPE, with cancer-associated fibroblasts serving as the primary source of CXCL16. Mechanistically, under the hypoxic conditions prevailing in the pleural cavity, cancer-associated fibroblasts in MPE undergo glycolysis, which in turn leads to an increase in the production of endogenous lactate. This elevated lactate induces histone H3 lysine 18 lactylation (H3K18la) at the promoter regions of both the CXCL16 gene and its transcription factor forkhead box O3 (FOXO3), which may contribute to CXCL16 transcription. TNFR2^+^ T_reg_ cells that express high levels of CXCR6, the only receptor for CXCL16, are subsequently recruited into MPE. The infiltration of TNFR2^+^ T_reg_ cells may reinforce the immunosuppressive milieu of MPE, facilitating disease progression. Collectively, these findings uncover a novel mechanism governing immunosuppression in MPE, providing new insights into potential therapeutic strategies to disrupt this process.

## Hypoxia induces Treg recruitment via lactate in tumors

Malignant pleural effusion (MPE) is a severe condition often seen in advanced lung cancer, characterized by the accumulation of fluid around the lungs due to the presence of cancer cells. Researchers explored why the immune system struggles to fight MPE. They focused on a type of immune cell known as TNFR2^+^ T_reg_ cells, which suppress the body’s ability to attack cancer. The study involved analyzing samples from patients and mice to understand how these cells are drawn to MPE. The researchers found that cancer-associated fibroblasts in MPE produce a protein called CXCL16, which attracts TNFR2^+^ T_reg_ cells. This process is driven by lactate, which regulates genes expression in a way that increases CXCL16 production. Blocking CXCL16 reduced the recruitment of these suppressive cells and slowed disease progression. The study suggests that targeting lactate production or CXCL16 could improve treatment outcomes for MPE.

This summary was initially drafted using artificial intelligence, then revised and fact-checked by the author.

## Introduction

Malignant pleural effusion (MPE) is a life-threatening complication commonly observed in advanced lung adenocarcinoma, resulting from the invasion of tumor cells into the pleura^1^. Due to the restricted treatment options, MPE advances rapidly, with a median survival period of just 5 months^2,3^. Despite studies highlighting the presence of antigen-presenting and effector cells within the immune landscape of MPE, the host immune system remains unable to control the malignancy^4,5^, probably attributed to the strong immunosuppressive activity^4^.

Regulatory T (T_reg_) cells play a prominent role in mediating immunosuppression by dampening the antitumor responses in MPE^6,7^. In particular, the subset of T_reg_ cells expressing tumor necrosis factor receptor 2 (TNFR2) is considered the most suppressive^8–13^. Our previous studies have shown that TNFR2^+^ T_reg_ cells are enriched in MPE and express high levels of immunosuppressive molecules, thereby promoting tumor progression^14,15^. Therefore, clarifying the mechanisms governing the accumulation of TNFR2^+^ T_reg_ cells is crucial for developing strategies to overcome the immunosuppression and improve clinical outcomes.

Chemokine-driven migration is a well-recognized mechanism underlying T_reg_ recruitment. Previous studies have reported that TNFR2^+^ T_reg_ cells express C–C motif chemokine receptor 4 (CCR4) and are recruited via C–C motif chemokine ligand 22 (CCL22) in ovarian cancer ascites^11^. Although a wide range of chemokines are elevated in MPE^16,17^, the specific chemotactic profiles that govern TNFR2^+^ T_reg_ migration remain unclear. Cancer-associated fibroblasts (CAFs) are well recognized as key producers of chemokines within the tumor microenvironment^18^. However, the roles of CAFs derived from MPE in establishing the chemotactic axis for TNFR2^+^ T_reg_ recruitment warrant further clarification.

Hypoxia, a hallmark of the tumor environment^19^, is known to induce chemokines expression in CAFs through hypoxia-inducible factor signaling^20^. Intriguingly, recent investigations have uncovered a more intricate mechanism. Lactate, a byproduct of glycolysis generated in response to hypoxia conditions, can modulate gene expression through a novel epigenetic modification known as lactylation^21–23^. In this context, we hypothesize that the hypoxic milieu of MPE may regulate chemokine expression in CAFs through histone lactylation, potentially contributing to the recruitment of TNFR2^+^ T_reg_ cells.

In this study, we show that CAFs in MPE undergo glycolysis, leading to an increase in endogenous lactate production. The elevated lactate induces histone H3 lysine 18 (H3K18) lactylation modification at the promoter regions of both the C–X–C motif chemokine ligand 16 (CXCL16) gene and its transcription factor forkhead box O3 (FOXO3), which may contribute to upregulate CXCL16 expression in CAFs. Consequently, TNFR2^+^ T_reg_ cells with high levels of C–X–C chemokine receptor type 6 (CXCR6), the exclusive receptor for CXCL16, may be preferentially recruited into MPE, thereby promoting its progression. These findings uncover a novel mechanism underlying the immunosuppression in MPE, offering new insights for developing therapeutic strategies aimed at disrupting this immunosuppressive process.

## Materials and methods

### Patients

MPE samples and peripheral blood (PB) were collected from patients diagnosed with lung adenocarcinoma at the Department of Respiratory and Critical Care Medicine, Union Hospital, Tongji Medical College (Wuhan, China) between May 2019 and May 2023. The diagnosis of MPE was confirmed through the identification of malignant cells in pleural fluid, closed pleural biopsy or both. Patients were excluded if they had undergone invasive procedures involving the pleural cavity or experienced chest trauma within the 3 months before hospitalization. At the time of sample collection, none of the patients had received any anticancer therapy, corticosteroids or nonsteroidal anti-inflammatory drugs. All specimens were obtained with informed patient consent, and the study was approved by the Wuhan Union Hospital Ethics Committee.

### Sample collection and processing

Fresh MPE samples (≥200 ml) and PB (5 ml) were collected within 24 h of hospitalization. The samples were centrifuged at 1,500 rpm for 10 min, after which the supernatants were aliquoted and stored at −80 °C for later chemokine analysis. The MPE cell pellets were resuspended in 1× phosphate-buffered saline (PBS) and pleural effusion mononuclear cells (PEMCs) were isolated by Ficoll-Hypaque gradient centrifugation (#18061, StemCell Technologies).

### Cell isolation and culture

To isolate CAFs, PEMCs were cultured in Dulbecco’s modified Eagle medium (DMEM) supplemented with 10% fetal bovine serum (FBS) and 100 U/ml penicillin–streptomycin for 12 h. Nonadherent cells were removed for subsequent experiments, while the remaining cells were cultured for 7–10 days. CAFs were then isolated using an Anti-Fibroblast MicroBeads Kit (#130-050-601, Miltenyi Biotec) and verified by flow cytometry (BD LSRFORTESSA X-20, BD Biosciences). Cells with >90% expression of fibroblast markers (FAP and CD29) and negative for endothelial (CD31) and epithelial cell markers (EPCAM and CD326) were classified as CAFs. Only CAFs with fewer than ten passages were used for further experiments. The purified CAFs were cultured in high-glucose DMEM containing 10% FBS, with the culture medium refreshed and collected every 2 days. The collected supernatant was used for lactate level determination and chemotaxis assay. To regulate the lactate production or lactylation, CAFs were treated with lactate (0–20 mM, #HY-B0389, MCE), glucose (0–20 mM, HY-B0389, MCE), sodium dichloroacetate (DCA, 0–20 mM, HY-Y0445A, MCE), oxamate (0–20 mM, HY-W013032A, MCE), rotenone (0–50 nM, HY-B1756, MCE) or β-alanine (0–20 mM, HY-N0230, MCE) for 48 h. Notably, CAFs receiving glucose or rotenone treatments were maintained in low-glucose DMEM throughout the experimental period. To assess the CXCL16 secretory capacity of fibroblasts, MRC-5 cells and isolated CAFs were seeded in 24-well plates at a density of 5 × 10^4^ cells per well in low-glucose DMEM supplemented with varying lactate concentrations (0–22 mM). Following 72-h incubation under standard culture conditions (37 °C, 5% CO₂), the culture media were collected, centrifuged (1,500*g*, 10 min) to remove cellular debris and stored at −80 °C until CXCL16 quantification.

Live TNFR2^+^CD4^+^ T cells and TNFR2^−^CD4^+^ T cells were isolated from MPE using fluorescence-activated cell sorting (FACS) with a BD FACSAria II Cell Sorter. After washing with 1× PBS, the cells were treated with TRIzol reagent (Thermo Fisher Scientific, #15596026CN) for subsequent RNA sequencing (RNA-seq) analysis. CD4^+^ T cells were purified from PEMCs with a CD4^+^ T cell isolation kit (#130-096-533, Miltenyi Biotec) according to the manufacturer’s instructions. The purity of each CD4^+^ T cell was measured by flow cytometry. A total of 1 × 10^6^ isolated CD4^+^ T cells were cultured in RPIM 1640 medium supplemented with 10% FBS, seeded into 48-well plates and treated with TNFR2 agonist (2.5 µg/ml, #HM2007BT, Hycult Biotech) or its isotype control (#HI4001, Hycult Biotech) for 48 h. Then, the cells were collected for further flow cytometry analysis.

Generally, all cells were cultured at 37 °C with 5% CO_2_. For experiments under hypoxic conditions, cells were grown in a specialized humidified chamber maintained at 1% oxygen, 94% nitrogen and 5% carbon dioxide for the specified duration.

### Cell lines

MRC-5 (#CCL-171, RRID: CVCL_0440) is a human fibroblast line cultured in Minimum Essential Medium supplemented with 10% FBS. NIH/3T3 (#CRL-1658, RRID: CVCL_0594) is a murine fibroblast line cultured in DMEM supplemented with 10% FBS. LLC (#CRL-1642, RRID: CVCL_4358) is a Lewis lung carcinoma cell line, and LLC-LUC (#CRL-1642-LUC2, RRID: CVCL_A4CM) is a luciferase-expressing variant of the LLC line, cultured in DMEM supplemented with 10% FBS. The mouse colon adenocarcinoma cell line MC-38 (#CL-0972, RRID: CVCL_B288) was purchased from the China Center for Type Culture Collection and cultured in DMEM supplemented with 10% FBS. All cells were obtained from the American Type Culture Collection.

### Murine MPE models

Six-week-old female C57BL/6 mice were purchased from Beijing Vital River Laboratory Animal Technology and fed in the specific-pathogen-free room in Tongji Medical College Animal Care Facility. To establish murine MPE models, 2 × 10^5^ LLC-LUC cells or MC-38 cells in 100 μl of 1× PBS were injected into the pleural cavity after anesthesia by 0.5% pentobarbital sodium. In addition, 1 × 10^5^ NIH/3T3 cells, LDHA-knockout NIH/3T3 cells or NC- NIH/3T3 cells were co-injected with 2 × 10^5^ LLC-LUC cells, respectively, for the indicated experiments. Bioluminescence imaging was performed on day 6 post-injection to confirm the successful establishment of MPE models. For the treatment study, normal drinking water was replaced with 200 ml of drinking water containing 1.2% β-alanine following LLC and NIH/3T3 cell co-injection. Oxamate (500 mg/kg, diluted in 1× PBS) was administered via intraperitoneal injection on day 6, day 8 and day 10 after co-injection. All mice were euthanized on day 12 for sample collection.

For in vivo CXCL16 neutralization experiments, anti-mouse CXCL16 antibody (50 μg/kg, #AF503, AB_2230043, RD) were injected into the pleural cavity on day 6, day 9 and day 12 after LLC-LUC cell injection. Mice were euthanized on day 14 for sample collection. To validate the roles of CXCL16 in vivo, recombinant mouse CXCL16 protein (50 μg/kg, #250-28-100UG, Thermo Fisher Scientific) was injected into the pleural cavity on day 6, day 8 and day 10 after LLC-LUC cell injection. Mice were euthanized on day 12 for further analysis. All animal experiments were approved by the Animal Care Committee of Tongji Medical College.

### Flow cytometry

To analyze cell subtypes of PEMCs, surface antibodies were added and incubated with the cells for 20 min at room temperature in the dark. Cells were then permeabilized using Fixation/Permeabilization Concentrate and Fixation/Perm Diluent (#00-5123-43, eBioscience) at a ratio of 1:3 for 30 min and then washed twice with 1× Permeabilization Buffer (#00-8333-56, eBioscience). Intracellular antibodies were subsequently added and incubated for 30 min in the dark. After staining, the cells were fixed with Fixation Buffer and analyzed by multicolor flow cytometry. Flow cytometry was performed on BD LSRFORTESSA X-20 (BD Biosciences), and the data were analyzed using FlowJo software, version 10.8.1 (Tree Star). The detailed information of antibodies used for flow cytometry are provided in Supplementary Table 1. The marker panels are listed in Supplementary Table 2.

### Transwell chemotaxis assay

A total of 1 × 10^6^ PEMCs isolated from patients with MPE or from MPE mice were uniformly seeded in the upper chamber of Transwell tissue culture inserts with an 8.0-μm pore diameter (Corning) and cultured in RPMI 1640 medium containing 0.5% FBS. For the chemotaxis assay, the lower chamber was filled with RPMI 1640 medium, MPE supernatant from patients, human CAF-culture supernatant, NC-NIH/3T3 cells culture supernatant or LDHA^−/−^ NIH/3T3 cell culture supernatant. In specific experimental conditions, the following reagents were added to the lower chamber: anti-CCL17 antibody (50 ng/ml, #MAB364-SP, R&D Systems), anti-CCL20 antibody (50 ng/ml, #MAB360-SP, R&D Systems), anti-CCL22 antibody (50 ng/ml, #MAB336-SP, R&D Systems), anti-CXCL16 antibody (50 ng/ml, #MAB976, R&D Systems) or recombinant human CXCL16 (100 ng/ml, #976-CX-025/CF, R&D Systems). The cells were incubated at 37 °C, 5% CO_2_ for 4 h. After incubation, transmigrated cells in the lower chambers were collected and analyzed by multicolor flow cytometry.

### Bioluminescence imaging

MPE mice were injected intraperitoneally with D-luciferin (150 mg/kg, MX4603-1G, MKBio) and rested for 10 min before anesthesia induction with 0.5% pentobarbital sodium. The anesthetized mice were imaged using the Bruker In Vivo FX PRO Imager. Luminescent images were captured with a 3-min exposure time, while reflectance images were taken with a 0.175-s exposure. Total photon flux was analyzed using Bruker MI SE software.

### Immunofluorescence

For immunofluorescence staining, human CAFs were isolated from MPE and seeded in 24-well culture plates containing coverslips. After 24 h when the cells had adopted an elongated spindle shape, they were fixed with 4% paraformaldehyde solution for 10 min. Following PBS washes, cells were permeabilized with 0.5% Triton X-100 for 5 min. Subsequently, the cells were blocked with 3% bovine serum albumin for 30 min, washed three times with PBS and incubated with primary antibodies overnight at 4 °C. After three additional washes with cold PBS, secondary antibodies were applied and cells were incubated at room temperature for 40 min, followed by three final washes with cold PBS. The coverslips were mounted with antifade mounting medium containing 4′,6-diamidino-2-phenylindole and imaged using confocal microscopy (Nikon).

### Human chemokine array

MRC-5 cells and CAFs were cultured with DMEM supplemented with 10% FBS for 48 h. The culture supernatants were collected, and chemokine levels were measured using the Human Chemokine Array Kit (#ARY017, R&D Systems) according to the manufacturer’s instructions.

### Enzyme-linked immunosorbent assay (ELISA)

The concentrations of CCL17, CCL20, CCL22 and CXCL16 were detected using Human CCL17 ELISA Kit (#NOV-NR-E10095-1x96T, Neobioscience), Human CCL20 ELISA Kit (#DM3A00, R&D Systems), Human CCL22 ELISA Kit (#NOV-NR-E10101-1x96T, Neobioscience), Human CXCL16 ELISA Kit (#EHC168, Neobioscience) and Mouse CXCL16 ELISA kit (#ELM-CXCL16, RayBiotech) according to the manufacturer’s instructions.

### Quantification of lactate levels

Lactate concentrations were quantified using the Lactate Colorimetric/Fluorometric Assay Kit (#ab65330, Abcam). The enzyme working solution and chromogenic agent were added to the samples. Following adding the reaction terminator, absorbance was measured at 530 nm using a multimode reader (PerkinElmer EnSpire). Lactate concentrations were calculated according to the manufacturer’s protocol.

### CCK-8 assay

CAFs were treated with DCA (0–20 mM), oxamate (0–20 mM) or retenone (0–50 nM) for 48 h. Cell viability was assessed using the Cell Counting Kit-8 (#C0038, Beyotime) according to the manufacturer’s instructions. Absorbance was measured at 450 nm using a Synergy 4 microplate reader (BioTek).

### Apoptosis assay

CAFs were treated with DCA (20 mM), oxamate (20 mM) or retenone (50 nM) for 48 h. Cell apoptosis was analyzed using the FITC Annexin V Apoptosis Detection Kit I (#556547, BD Biosciences) following the manufacturer’s protocol. Data were acquired and processed using FlowJo software (v10.8.1, BD Life Sciences).

### Vector construction and cell transfection

The human FOXO3 overexpression plasmid (pCMV-FOXO3(human)-FLAG-Neo, #P38078), short hairpin RNAs (shRNAs) targeting human FOXO3 (pLKO.1-U6-FOXO3(human)-shRNA1-PGK-Puro, #P21814) and shRNAs targeting human LDHA (pML-LKO.1-U6-LDHA(human)-shRNA1-hPGK-Puro, #P27303； pML-LKO.1-U6-LDHA(human)-shRNA2-hPGK-Puro, #P27302; pML-LKO.1-U6-LDHA(human)-shRNA3-hPGK-Puro, #P27304) were purchased from MiaoLingPlasmid. The sequences of plasmids are available at http://www.miaolingbio.com/. Corresponding empty vectors were used as the control. Transfection was performed using Lipofectamine 3000 (#L3000015, Thermo Fisher Scientific) according to the manufacturer’s instructions.

CRISPR–Cas9 technology was used to knock out the LDHA gene (LDHA-KO) in NIH/3T3 fibroblasts. In brief, single guide RNAs (sgRNAs) targeting the mouse LDHA gene were designed using ChopChop (http://chopchop.cbu.uib.no) and synthesized by TSINGKE. The sgRNAs were annealed by heating at 95 °C for 5 min, followed by gradual cooling to stabilize secondary structures. These sgRNAs were cloned into the pX459 vector, and stable transfectants were selected using puromycin (#A1113803, Thermo Fisher Scientific). The sequences of the sgRNAs used in this study are listed in Supplementary Table 3.

### Chromatin immunoprecipitation and qPCR (ChIP–qPCR) assays

ChIP assays were performed using the Simple ChIP Enzymatic Chromatin IP Kit (#9003, Cell Signaling Technology) following the manufacturer’s instructions. Anti-L-lactyl-histone H3 (Lys18) rabbit mAb (#PTM-1427RM, PTM BIO) and anti-FOXO3 antibody (#10849-1-AP, Proteintech) were used for immunoprecipitation. The precipitated DNA fragments were quantified by qPCR, and the results are presented as percent input. FOXO3 binding sites on CXCL16 promoters were predicted using the JASPAR database (http://jaspar.genereg.net). The primer sequences used for ChIP–qPCR are provided in Supplementary Table 4.

### Cleavage under targets and tagmentation (CUT&Tag) assay

The CUT&Tag assay was performed using a commercial kit (#TD903, Vazyme) according to the manufacturer’s protocol. In brief, a total of 60,000 cells were collected, washed and incubated with magnetic beads coated with Concanavalin A. Following this, the cells were incubated with anti-L-lactyl-histone H3 (Lys18) rabbit mAb (#PTM-1427RM, AB_3076698, PTM BIO) for 2 h, then incubated for an additional hour with a secondary antibody at room temperature. Next, tagmentation was initiated using the hyperactive pG-Tn5 transposonase, after which the reaction was terminated. DNA fragments were extracted using phenol–chloroform–isoamyl alcohol. These fragments were amplified via PCR using indexed P5 and P7 primers. The final library products were enriched, quantified and sequenced on a NovaSeq 6000 sequencer (Illumina) in PE150 mode.

### Dual-luciferase reporter assay

The CXCL16 promoter region (−1,500 to 100 bp) was synthesized by TSINGKE and cloned into pGL3-Basic vector (Promega) to construct firefly luciferase reporters. Purified CAFs were co-transfected with the CXCL16 promoter reporter plasmids and a FOXO3 overexpression plasmid both in the pGL3-Basic vector. The Renilla luciferase reporter vectors pRL-TK were used as internal control. After 48 h of transfection, firefly and Renilla luciferase activities were measured using the Dual-Luciferase Reporter Assay System (#E1910, Promega) according to the manufacturer’s instructions. The sequences used for luciferase reporter assay are provided in Supplementary Table 5.

### Western blot

Whole-cell lysates were prepared using RIPA buffer supplemented a protease inhibitor cocktail (1:100, #P1005, Beyotime Biotechnology). Protein concentrations were quantified using the BCA Protein Assay Kit (#P0012, Beyotime Biotechnology). Equal amounts of protein were mixed with 1× loading buffer and boiled at 95 °C for 15 min. Proteins were separated by sodium dodecyl sulfate–polyacrylamide gel electrophoresis and transferred onto a polyvinylidene difluoride membrane on ice. The membrane was blocked with 5% skim milk in Tris-buffered saline containing 0.05% Tween 20 (TBST) at room temperature for 2 h, followed by overnight incubation with primary antibodies at 4 °C, including anti-Pan-Kla antibody (#PTM-1401, PTM BIO), anti-H3K18la antibody (#PTM-1406RM, PTM BIO), anti-H3K9la antibody (#PTM-1419RM, PTM BIO), anti-H3K27la antibody (#PTM-1428, PTM BIO), anti-H4K12la antibody (#PTM-1411RM, PTM BIO), anti-H4K8la antibody (#PTM-1415RM, PTM BIO), anti-CXCL16 antibody (#60123-1-Ig, Proteintech), anti-FOXO3 antibody(#10849-1-AP, Proteintech), anti-GAPDH antibody (#60004-1-Ig, Proteintech) and anti-histone H3 antibody (#17168-1-AP, Proteintech). The membranes were washed three times with TBST at room temperature for 15 min each and then incubated with secondary antibodies for 1 h at room temperature. After three additional washes with TBST, chemiluminescent substrate solution (#E423-01, Vazyme) was applied, and the signal was detected using the UVP chemiluminescence imaging system.

### RNA-seq analysis

Total RNA was extracted from TNFR2^+^CD4^+^ T cells and TNFR2^−^CD4^+^ T cells from three MPE specimens using TRIzol reagent (Invitrogen) according to the manufacturer’s protocol. The RNA quality was determined by 5300 Bioanalyser (Agilent) and quantified using the ND-2000 (NanoDrop Technologies). Only high-quality RNA samples (optical density (OD)_260/280_ ≈ 1.8–2.2, OD_260/230_ ≥ 2.0, RNA Quality Number (RQN) ≥ 6.5, 28S:18S ≥ 1.0, >1 μg) were used to construct the sequencing library. RNA purification, reverse transcription, library construction and sequencing were performed at Shanghai Majorbio Bio-pharm Biotechnology according to the manufacturer’s protocol. Libraries were prepared using the Illumina TruSeq RNA Sample Preparation Kit, and sequencing was carried out on an Illumina NovaSeq 6000 platform with a read depth of 20 million reads per sample. Differential gene expression analysis between TNFR2^+^CD4^+^ T cells and TNFR2^−^CD4^+^ T cells was conducted using the DESeq2 pipeline, with an adjusted *P* value (false discovery rate) threshold of <0.05 to identify significantly differentially expressed genes (DEGs). Raw sequencing data were analyzed on the online platform of Majorbio Cloud Platform (www.majorbio.com)^24^. The raw data generated in this study have been uploaded to Sequence Read Archive (https://www.ncbi.nlm.nih.gov/sra/), BioProject ID: PRJNA1170877.

The normalized RNA-seq expression data (log_2_(transcripts per million [TPM] + 0.001) transformed) for CXCL16, FOXP3 and TNFR2, along with overall survival information, were obtained from The Cancer Genome Atlas (TCGA) via the UCSC Xena browser (https://xenabrowser.net/). To assess the prognostic relevance of CXCL16, patients were divided into high and low CXCL16 expression groups based on the optimal cutoff value determined using the surv_cutpoint function from the survminer package (V.0.4.9), with minprop = 0.25 to ensure sufficient sample size in both groups. Kaplan–Meier survival curves were generated using the survival (V.3.8-3) and survminer packages and visualized with ggsurvplot function. To assess the association between CXCL16, TNFR2 and FOXP3 across different cancer types, correlation analysis was conducted using the corr.test function from the psych package(V.2.4.3) and visualized in a heatmap-style dot plot, where dot size and color intensity reflect the strength of correlation and color hue indicates its direction.

### Single-cell RNA-seq analysis

Single-cell RNA-seq analysis of CXCL16^+^ CAFs and CXCL16^-^ CAFs was conducted using previously published data (PRJNA970083)^25^. The Seurat R package (v4.2.0) was used for primary analysis. Low-quality cells were excluded based on established criteria from prior research. Data normalization was performed with a scaling factor of 10,000, and the top 2,000 variable genes were identified using the FindVariableFeatures function. Principal component analysis was then applied, using the first 30 principal components in the FindNeighbors algorithm to identify cell clusters with the FindClusters function. Cluster visualization was achieved through *t*-distributed stochastic neighbor embedding. DEGs within clusters were identified with the FindAllMarkers function. Enrichment analysis was performed using the clusterProfiler package (v4.2.0) and gene set variation analysis (GSVA) with the GSVA package (v1.42.0), using gene sets from the MSigDB database.

### Statistical analysis

Statistical significance was assessed using two-tailed Student’s *t*-test, Mann–Whitney test, Wilcoxon test, one-way analysis of variance (ANOVA) and Pearson or Spearman correlation coefficient. Survival time was analyzed with the Kaplan–Meier method and compared using the log-rank test. Data are presented as mean ± s.d., with a *P* value <0.05 considered statistically significant. Significance levels are indicated as follows: *P < 0.05, **P < 0.01, ***P < 0.001, ****P < 0.0001, and ‘ns’ for not significant. All analyses were conducted using Prism V8.1.2 (GraphPad Software).

## Results

### CXCL16–CXCR6 axis mediates the recruitment of TNFR2^+^ T_reg_ cells in MPE

**Fig. 1:**
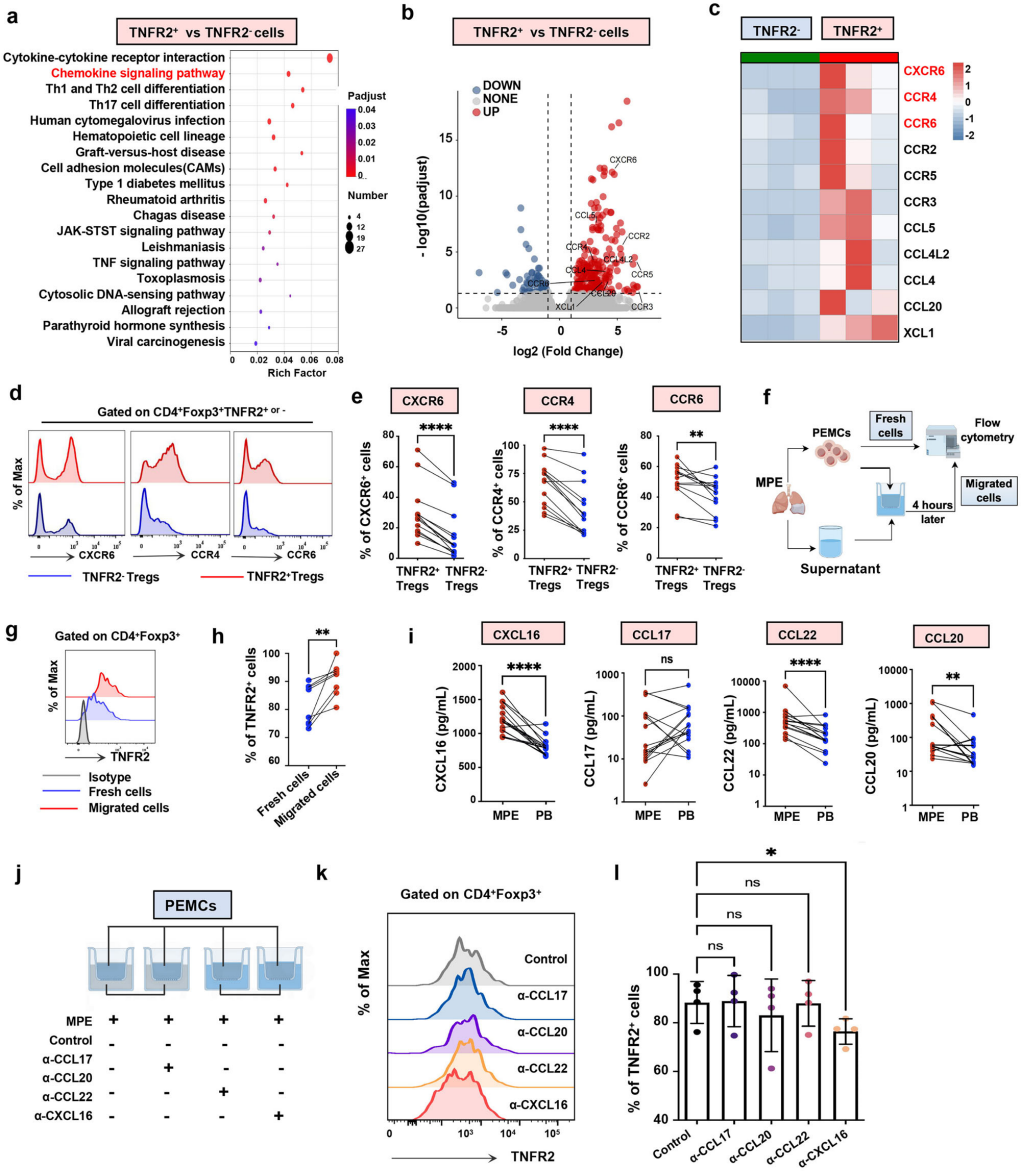
CXCL16–CXCR6 axis mediates the recruitment of TNFR2^+^ T_reg_ cells to MPE. **a** KEGG pathway enrichment of DEGs between FACS-sorted TNFR2^+^and TNFR2^−^CD4^+^ T cells from MPE (*n* = 3). **b**, **c** Volcano plot (**b**) and heatmap (**c**) of DEGs related to the chemokine signaling pathway. **d**, **e** Flow cytometry histograms (**d**) and comparisons of chemokine receptor expression (CXCR6, CCR4 and CCR6) (**e**) on TNFR2^+^ T_reg_ cells and TNFR2^−^ T_reg_ cells (*n* = 13). **f** Schematic of Transwell chemotaxis assay testing TNFR2^−^ T_reg_ chemotaxis toward MPE supernatant (by Figdraw). **g**, **h** Transwell chemotaxis assay comparing TNFR2^+^ T_reg_ frequencies between freshly isolated cells and cells that migrated toward MPE supernatant after 4 h incubation. **i** Concentrations of CXCL16, CCL17, CCL22 and CCL20 in MPE and PB were quantified using ELISA (*n* = 16). **j** Schematic of chemotaxis assay to investigate the chemotatic axis to attract TNFR2^+^ T_reg_ cells in MPE. **k**, **l** Flow cytometry histograms and comparisons of chemotaxis of TNFR2^+^ T_reg_ cells in response to MPE in the presence of anti-CXCL16, anti-CCL17, anti-CCL22 or anti-CCL20 mAbs. Data shown in **d**, **e**, **g**–**i**, **k** and **l** are representative of at least three independent experiments (mean ± s.d.). Statistical analysis was performed using paired two-tailed Student’s *t*-test (**e** and **h**), Wilcoxon test (**i**) or one-way ANOVA (**l**). *P < 0.05, **P < 0.01, ****P < 0.0001. ns not significant, mAbs monoclonal antibodies.

To investigate the chemotactic profile of TNFR2^+^ T_reg_ cells in MPE, RNA-seq was performed on TNFR2^+^CD4^+^ T cells and TNFR2^−^CD4^+^ T cells isolated from three MPE specimens. Kyoto Encyclopedia of Genes and Genomes (KEGG) pathway analysis indicated that TNFR2 expression mainly impacted the cytokine–cytokine receptor interaction and chemokine signaling pathway (Fig. 1a). Notably, chemokines represent a specialized subset of cytokines primarily responsible for guiding cell migration, particularly T cell recruitment^26,27^, suggesting that TNFR2 expression may enhance T_reg_ migratory capacity. Among the chemokine receptors, CXCR6, CCR4 and CCR6 showed significantly higher expression in TNFR2^+^ T_reg_ cells compared with TNFR2^−^ T_reg_ cells, further confirmed by flow cytometry (Fig. 1b–e and Supplementary Fig. 1a). In vitro chemotaxis assays showed that TNFR2^+^ T_reg_ cells displayed greater chemotactic activity toward MPE than TNFR2^−^ T_reg_ cells (Fig. 1f–h). Furthermore, we noted that TNFR2 signaling activation specifically increased CXCR6 expression (Supplementary Fig. 1b, c) without affecting CCR4 or CCR6 levels (Supplementary Fig. 1d–g). Correlation analysis further revealed a strong positive correlation between TNFR2 and CXCR6 expression levels (*r* = 0.8213), rather than CCR4 (*r* = −0.2591) or CCR6 (*r* = 0.3364) (Supplementary Fig. 1h–k). These findings suggest that the enhanced migration of TNFR2^+^ T_reg_ cells may be dependent on the CXCR6-mediated chemotactic axis. To confirm this hypothesis, ELISA measured the concentrations of CXCL16 (ligand for CXCR6), CCL17, CCL22 (ligands for CCR4) and CCL20 (ligands for CCR6) in MPE. The results showed that CXCL16, CCL22 and CCL20 were elevated in MPE compared with PB (Fig. 1i). Notably, CXCL16 exhibited consistently elevated levels across patients with MPE, with minimal interindividual variability (interquartile range (IQR) 1,044–1,369 pg/ml). By contrast, CCL20, and CCL22 displayed marked interpatient heterogeneity, with broader ranges of expression (CCL20: IQR 42.33–408 pg/ml; CCL22: IQR 336–828 pg/ml) (Fig. 1i). Consistent with this findings, ex vivo chemotactic assay (Fig. 1j) revealed that blocking CXCL16 significantly impaired TNFR2^+^ T_reg_ migration toward MPE (Fig. 1k, l), highlighting the critical role of the CXCL16–CXCR6 axis in mediating TNFR2^+^ T_reg_ recruitment.

### CAFs are the major source of CXCL16 in MPE

**Fig. 2:**
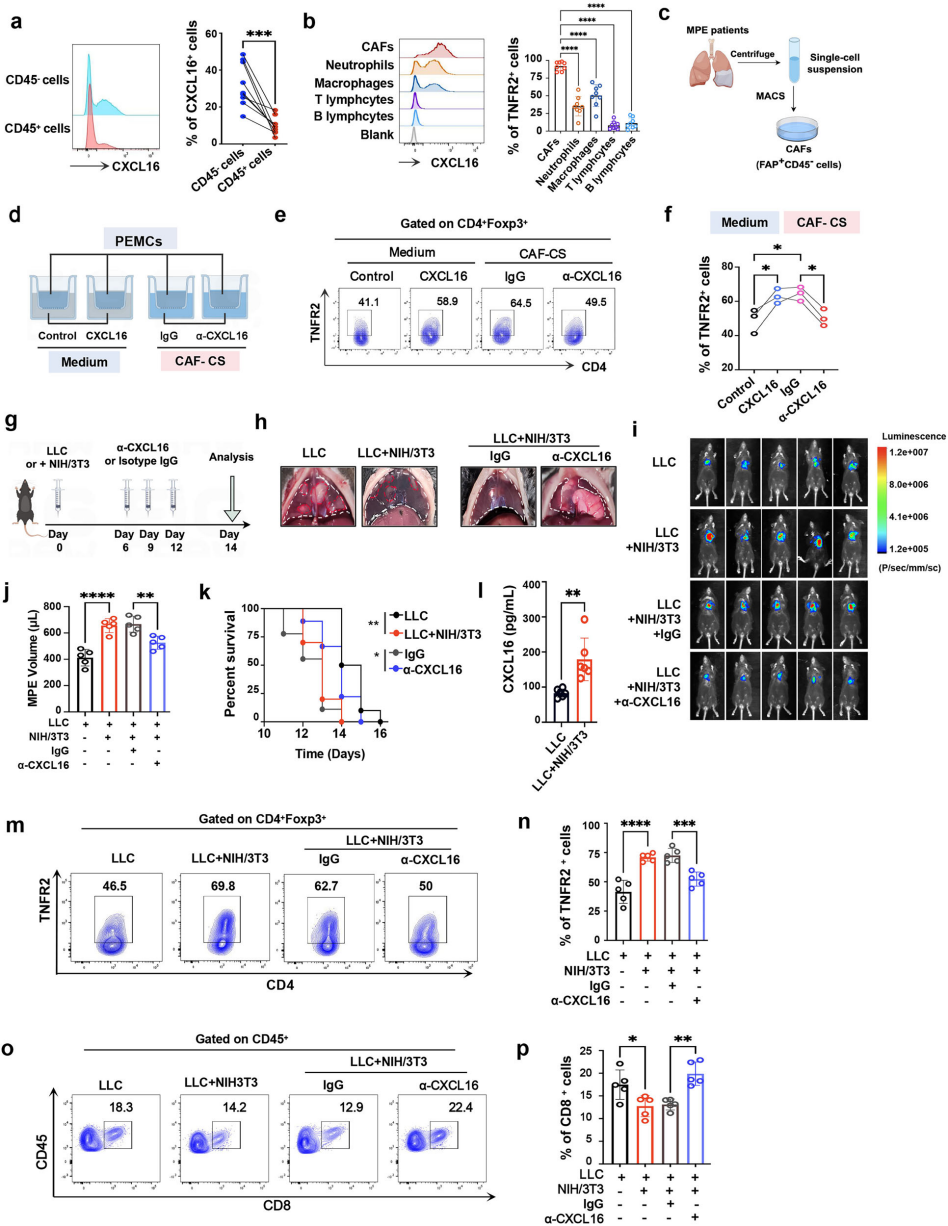
CAF-derived CXCL16 promotes TNFR2^+^ T_reg_ recruitment and drives MPE progression. **a** Flow cytometry analysis comparing of CXCL16 expression on CD45^+^ and CD45^−^ cells from MPE (*n* = 9). **b** CXCL16 expression on major cell populations of MPE: CAFs (CD45^−^FAP^+^), neutrophils (CD45^+^CD11b^+^Ly6G^+^), macrophages (CD45^+^CD68^+^), T lymphocytes (CD45^+^CD3^+^) and B lymphocytes (CD45^+^CD19^+^) from MPE (*n* = 8). **c** Schematic of CAF purification from MPE using MACS (by Figdraw). **d** Schematic of Transwell assay testing TNFR2^+^ T_reg_ chemotaxis toward CXCL16-supplemented medium or CAF culture supernatant, with or without anti-CXCL16 mAbs (by Figdraw). **e**, **f** Representative flow cytometry plots (**e**) and comparisons (**f**) of TNFR2^+^ T_reg_ frequencies recruited under different conditions (*n* = 3). **g** Schematic diagram of the MPE mouse model. **h** Representative images showing mouse MPE and pleural cavity tumors. **i**–**k** Bioluminescence images depicting the growth (*n* = 5) (**i**), MPE volume (*n* = 5) (**j**) and Kaplan–Meier survival plot (**k**) of MPE mice (*n* = 9–10 per group). **l** Concentrations of CXCL16 in MPE from mouse models (*n* = 6). **m**, **n** Frequencies of TNFR2^+^ cells among T_reg_ cells in murine MPE of each group. **o**, **p** Frequencies of CD8^+^ T cells from mouse MPE in each group. Data shown in **a**, **b**, **e**, **f** and **h**–**p** are representative of at least three independent experiments (mean ± s.d.). Statistical analysis was performed using paired two-tailed Student’s *t*-test (**a**), unpaired two-tailed Student’s *t*-test (**l**), one-way ANOVA (**b**, **f**, **j**, **n** and **p**) or log-rank test (**k**). *P < 0.05, **P < 0.01, ***P < 0.001, ****P < 0.0001. ns not significant, CS culture supernatant, MACS magnetic-activated cell sorting, α-CXCL16 CXCL16 neutralizing antibody.

To further identify the source of CXCL16 in MPE, we performed flow cytometric analysis on major cell populations isolated from MPE samples. The results revealed that approximately 80% of CXCL16^+^ cells were CD45^−^ cells (Fig. 2a). In addition, the proportion of CXCL16^+^ cells within CAFs (defined as CD45^−^FAP^+^) surpassed 90%, a proportion significantly higher than other populations, including neutrophils (CD45^+^CD11b^+^Ly6G^+^; 35.29 ± 13.79%), macrophages (CD45^+^CD68^+^; 50.25 ± 15.59%), T cells (CD45^+^CD3^+^; 8.15 ± 5.68%) and B cells (CD45^+^CD19^+^; 11.28 ± 7.5%) (Fig. 2b and Supplementary Fig. 2a).These results indicate that CAFs are the predominant producers of CXCL16 in MPE.

To assess whether CAF contributes to TNFR2^+^ T_reg_ recruitment, we isolated CAFs from MPE (Fig. 2c and Supplementary Fig. 2b–d) and collected their culture supernatant for chemotactic assay (Fig. 2d). The results showed that the culture supernatant of CAFs, enriched with various chemokines (Supplementary Fig. 2e, f), effectively attracted TNFR2^+^ T_reg_ cells (Fig. 2e, f). Importantly, this effect was markedly diminished after CXCL16 blockade (Fig. 2e, f), suggesting a central role of CAF-derived CXCL16 in facilitating TNFR2^+^ T_reg_ migration.

### CAFs-derived CXCL16 promotes TNFR2^+^ T_reg_ accumulation and MPE progression

To investigate the impact of CAF-derived CXCL16 in MPE progression in vivo, we used a murine MPE model using Lewis lung carcinoma cells (LLCs) (Fig. 2g). NIH/3T3 fibroblasts, a normal mouse fibroblast cell line that initially expresses low levels of CXCL16, markedly upregulated CXCL16 upon exposure to the culture supernatant of LLCs (Supplementary Fig. 2g), suggesting their transition toward a CAF-like characteristics within the MPE environment. As expected, intrapleural injection of NIH/3T3 fibroblasts alone did not induce MPE formation (Supplementary Fig. 2h). By contrast, co-injection with LLCs significantly promoted MPE development, as indicated by increased MPE volume (Fig. 2h–j) and shortened survival time (Fig. 2k). ELISA assays further revealed that the presence of fibroblasts significantly elevated CXCL16 levels in MPE (Fig. 2l). Notably, blocking CXCL16 substantially reduced MPE volume while prolonging the survival time (Fig. 2h–k). These data suggest that CAF-derived CXCL16 plays a critical role in MPE progression.

We next evaluated the immune landscape of MPE. The presence of NIH/3T3 fibroblasts significantly increased the proportion of TNFR2^+^ T_reg_ cells in MPE (Fig. 2m, n and Supplementary Fig. 3a). By contrast, the proportion of CD8^+^ T cells, as key effectors in the antitumor immunity, was significantly decreased (Fig. 2o, p and Supplementary Fig. 3b). In addition, the cytotoxicity of CD8^+^ T cells was markedly impaired, as shown by reduced proportions of IFN-γ^+^ cells (Supplementary Fig. 3c, d), granzyme B^+^ cells (Supplementary Fig. 3e, f) and perforin^+^ cells (Supplementary Fig. 3g, h). Importantly, these immunosuppressive effects were reversed after CXCL16 blockade (Fig. 2m–p and Supplementary Fig. 3c–h), indicating a role for CXCL16 in shaping the local immune environment.

To assess the generalizability of this mechanism, we established a colorectal cancer-derived MPE model, using MC-38 cells^28^ (Supplementary Fig. 4a). Similar to the LLCs model, co-injection of MC-38 cells with NIH/3T3 fibroblasts enhanced MPE burden and mortality (Supplementary Fig. 4b, c), accompanied by elevated TNFR2^+^ T_reg_ proportions and impaired CD8^+^ T cell function, all of which were attenuated by CXCL16 blockade (Supplementary Fig. 4d–i). Clinical analysis of TCGA datasets revealed that elevated CXCL16 expression correlated with reduced overall survival (Supplementary Fig. 5a–h) and showed positive associations with Foxp3 and TNFR2 (Supplementary Fig. 5i) across multiple malignancies. These results indicate CAF-derived CXCL16 as a critical regulator of TNFR2^+^ T_reg_ accumulation and MPE progression, with conserved tumor-type effects suggesting suggesting broader implications in malignant progression.

### Lactate production is associated with high expression of CXCL16 in CAFs

**Fig. 3:**
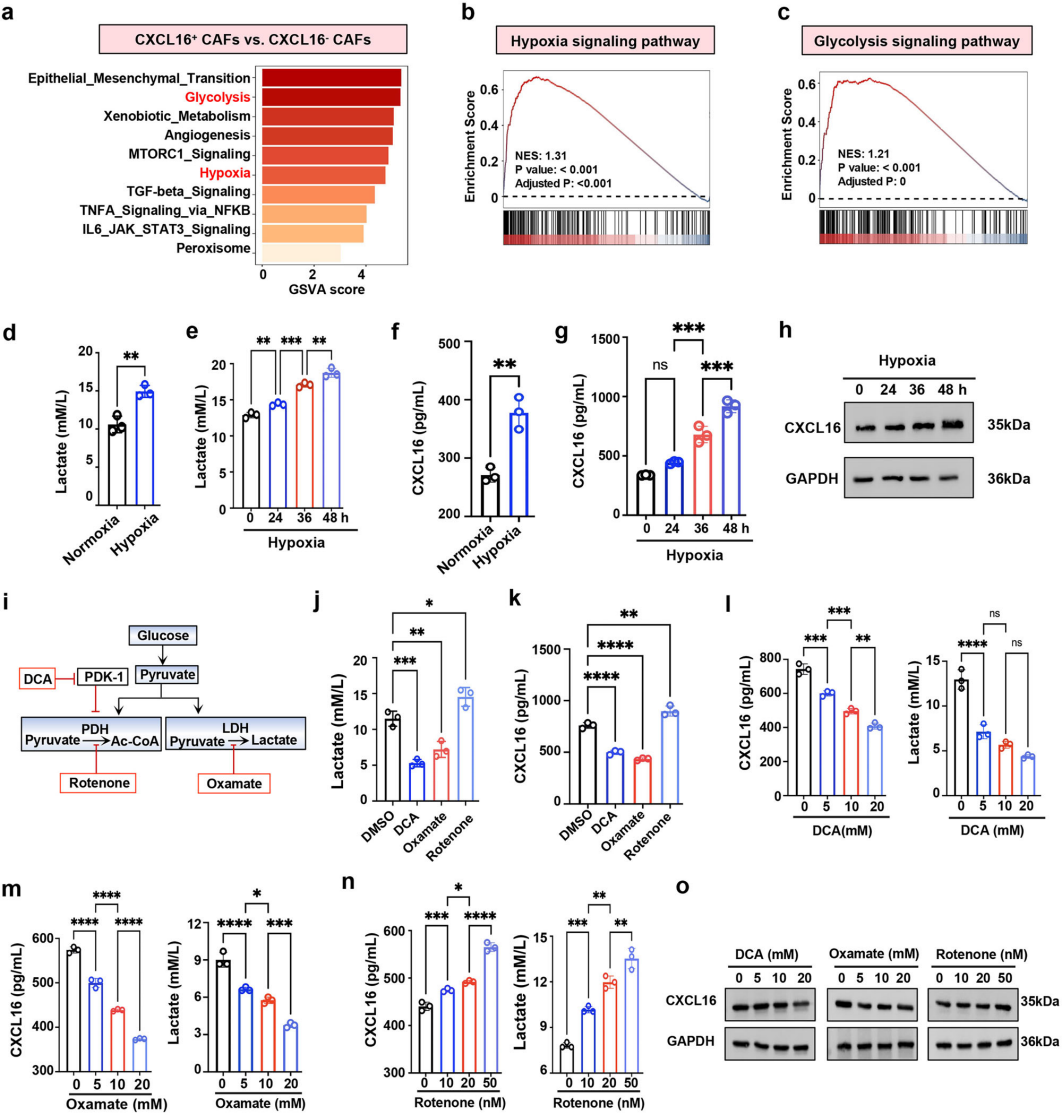
Hypoxia-induced lactate production upregulates CXCL16 expression in CAFs. **a** GSVA of signaling pathways enriched in CXCL16^+^ CAFs compared with CXCL16^−^ CAFs. **b**, **c** Gene set enrichment analysis (GSEA) was performed on gene sets related to the hypoxia signaling pathway (**b**) and the glycolysis signaling pathway (**c**). A positive NES indicates higher expression in CXCL16^+^ CAFs. **d**, **e** Lactate levels in CAFs cultured under normoxia (21% oxygen) or hypoxia (1% oxygen) for 48 h or at indicated time points (*n* = 3). **f**, **g** CXCL16 concentrations in CAFs supernatant under normoxia or hypoxia for 48 h or at indicated time points (*n* = 3). **h** Western blot of CXCL16 expression in CAFs under hypoxia at designated times. **i** Schematic of metabolic modulators targeting glycolysis or lactate production. **j**, **k** Lactate levels (**j**) and CXCL16 concentrations (**k**) in CAFs treated with indicated glycolysis modulators for 48 h (*n* = 3). **l**–**n** Dose-dependent effects of DCA (**l**), oxamate (**m**) or rotenone (**n**) on lactate and CXCL16 levels in CAFs supernatant. **o** Western blot analysis of CXCL16 expression in CAFs treated with the indicated concentrations of glycolysis modulators for 48 h. Data shown in **d**–**h** and **j**–**o** are representative of at least three independent experiments (mean ± s.d.). Statistical analysis was performed using unpaired two-tailed Student’s *t*-test (**d** and **f**) or one-way ANOVA (**e**, **g** and **j**–**n**). *P < 0.05, **P < 0.01, ***P < 0.001, ****P < 0.0001. ns not significant, NES normalized enrichment score, DMSO dimethylsulfoxide, DCA dichloroacetate.

To investigate the mechanism underlying elevated CXCL16 expression in CAFs, single-cell RNA-seq analysis were performed on CXCL16^+^ CAFs and CXCL16^−^ CAFs using previously published MPE data^25^. The results revealed the enrichment of glycolysis and hypoxia signaling pathway in CXC16^+^ CAFs (Fig. 3a–c). In vitro, CAFs cultured under hypoxic conditions exhibited a time-dependent increase in lactate production (Fig. 3d, e), accompanied by a parallel increase in CXCL16 expression in CAFs over time (Fig. 3f–h). These findings suggest a potential link between glycolysis, lactate production and CXCL16 expression in CAFs.

To further clarify the relationship, we tested whether glycolytic enzymes that modulate lactate levels influenced CXCL16 expression. Our findings showed that treatment of CAFs with DCA and oxamate by targeting pyruvate dehydrogenase and lactate dehydrogenase (LDH) (Fig. 3i) led to reduced lactate production (Fig. 3j) and a concomitant decrease in CXCL16 expression (Fig. 3k) in a dose-dependent manner (Fig. 3l, m). Conversely, disrupting oxidative phosphorylation with rotenone, the classical inhibitor of mitochondrial complex I^29^, enhanced glycolysis (Fig. 3i), led to dose-dependent increases in both lactate and CXCL16 levels (Fig. 3j, k, n, o). Overall, these observations demonstrate a positive correlation between lactate production and CXCL16 expression, suggesting a role of glycolysis-driven lactate production in modulating CXCL16 expression in CAFs.

### Endogenous lactate production promotes CXCL16 expression in CAFs via histone H3K18 lactylation

Lactate, once considered as a byproduct of glycolysis, is now recognized for its role in modifying lysine residues, leading to a novel epigenetic change known as lactylation^21^. In the study, we observed elevated lactate levels in MPE (Supplementary Fig. 6a), accompanied by widespread histone lactylation at multiple sites in CAFs, including histone H3K18, H3K9, H3K14, H3K27, H4K12 and H4K8 (Supplementary Fig. 6b). Inhibition of lactate production via oxamate or LDHA knockdown treatment markedly reduced these modifications (Supplementary Fig. 6b). Among these, lactylation at histone H3K18 (H3K18la) has been reported to stimulate gene expression^21,30,31^. Based on H3K18la levels (Supplementary Fig. 6c, d), patients with MPE were categorized into H3K18la^high^ and H3K18la^low^ groups (Supplementary Table 6). The high-H3K18la group exhibited greater tumor cell infiltration (Supplementary Fig. 6e), larger MPE volumes (Supplementary Fig. 6f) and worse performance status (Supplementary Fig. 6g). This suggests that H3K18 lactylation in CAFs may predict poor prognosis in patients with MPE. Interestingly, patients in the high H3K18la group also displayed elevated levels of CXCL16 (Supplementary Fig. 6h) and a higher proportion of TNFR2^+^ T_reg_ cells (Supplementary Fig. 6i), suggesting the potential role of H3K18la in increasing CXCL16 expression, thereby promoting the immunosuppression in MPE.

**Fig. 4:**
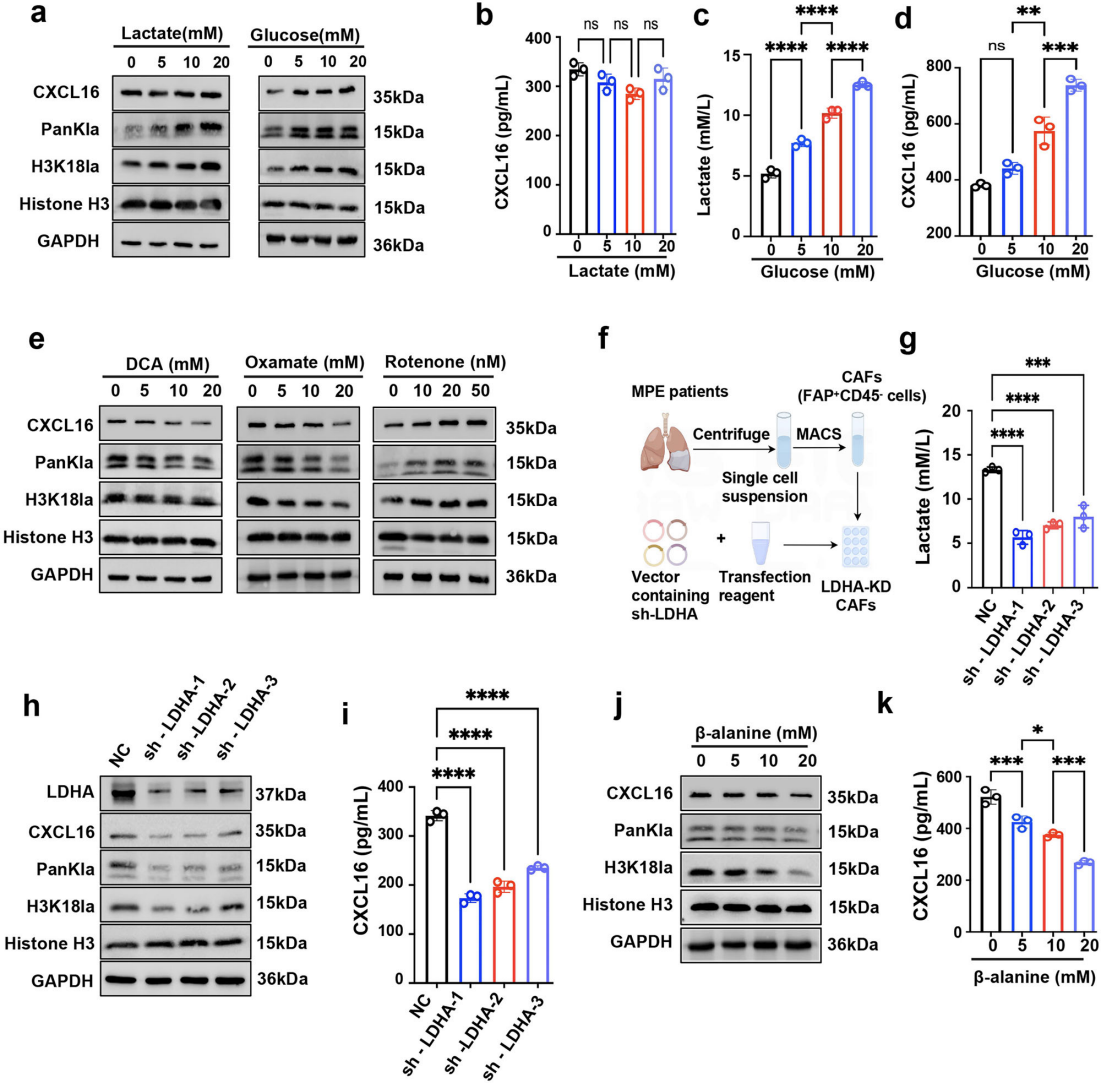
Endogenous lactate-induced histone lactylation increases CXCL16 expression of CAFs. **a** Western blot analysis of the indicated proteins in CAFs treated with increasing concentrations of exogenous lactate or glucose for 48 h. **b** CXCL16 concentrations in CAFs supernatant after exogenous lactate treatment. **c**, **d** Lactate (**c**) and CXCL16 (**d**) levels in CAFs supernatant after treatment with glucose for 48 h. **e** Western blot analysis of the indicated proteins in CAFs treated with DCA, oxamate or rotenone for 48 h. **f** Schematic of LDHA knockdown in CAFs. **g** Lactate in the culture supernatant of LDHA-knockdown CAFs. **h** Western blot analysis of the indicated proteins in LDHA-knockdown CAFs. **i** CXCL16 levels in the supernatant of LDHA-knockdown CAFs. **j** Western blot analysis of the indicated proteins in CAFs treated with increasing concentrations of β-alanine, lactylation modulator, for 48 h. **k** CXCL16 concentrations in CAFs supernatant after β-alanine treatment. Data shown in **a**–**e** and **g**–**k** are representative of at least three independent experiments (mean ± s.d.). Statistical analysis was performed using one-way ANOVA (**b**–**d**, **g**, **i** and **k**). *P < 0.05, **P < 0.01, ***P < 0.001, ****P < 0.0001. ns not significant, KD knockdown, sh-LDHA shRNA against LDHA, sh-NC negative control.

To further investigate the causality, exogenous lactate was added to CAF cultures. Intrestingly, H3K18la levels obviously increased (Fig. 4a), while CXCL16 expression remained relatively unchanged (Fig. 4a, b). This discrepancy may be attributed to the elevated lactate levels already present in CAFs. Supporting this, normal fibroblasts exhibited dose-dependent increases until a plateau was reached beyond 10 mM (Supplementary Fig. 6j), a concentration substantially lower than CAF supernatant levels (13.8 ± 3.08 mM; Supplementary Fig. 6a). These findings suggest that CXCL16 expression is lactate sensitive but saturable, and further increases in exogenous lactate have limited effect once this threshold is exceeded. By contrast, H3K18la continued to accumulate even after CXCL16 plateaued, implying additional lactylation-dependent gene regulation beyond CXCL16. The broader implications of H3K18la in CAFs warrant further investigation.

Given the strong correlation between lactate production and CXCL16 expression, we further investigated the role of endogenous lactate in CAFs under glucose-limited conditions. In the absence of glucose, CAFs secreted low levels of lactate (5.2 ± 0.36 mM; Fig. 4c), as well as CXCL16 (Fig. 4d). As glucose concentrations increase, lactate production significantly increased (Fig. 4c), along with dose-dependent elevations in H3K18la and CXCL16 levels (Fig. 4a, d). Consistently, inhibition of lactate production with DCA and oxamate reduced both H3K18la levels and CXCL16 expression, while treatment with rotenone had the opposite effect (Fig. 4e). To ensure that these metabolic interventions did not introduce confounding cytotoxic effects, we performed CCK-8 assays and Annexin V/PI staining to assess cell viability and apoptosis under the same treatment conditions. The results showed that DCA, oxamate and rotenone did not significantly impair cell viability (Supplementary Fig. 7a) or induce apoptosis in CAFs (Supplementary Fig. 7b, c), supporting the specificity of the metabolic effects observed.

In addition, we constructed three plasmid vectors encoding shRNA against LDHA (sh-LDHA-1, sh-LDHA-2 and sh-LDHA-3) (Fig. 4f) to reduce endogenous lactate production in CAFs (Fig. 4g). All three constructs showed varying degrees of knockdown efficiency (Fig. 4g, h). The results showed that knockdown of LDHA resulted in a marked decrease in both H3K18la levels and CXCL16 expression (Fig. 4h, i), reinforcing the role of endogenous lactate of CAFs in H3K18la and CXCL16 expression.

Alanyl-tRNA synthetase (AARS1) has been identified as a lactylation writer enzyme that catalyzes the addition of lactyl groups to lysine residues in a lactate-dependent manner^32–34^. When CAFs were treated with β-alanine, a structural analog of lactate that disrupts lactate binding to AARS1^32^, H3K18la levels notably decreased (Fig. 4j), accompanied by a dose-dependent reduction in CXCL16 expression (Fig. 4j, k). These findings suggest that endogenous lactate in CAFs may promote CXCL16 expression via histone H3K18 lactylation.

### H3K18la enrichment in the promoter regions of FOXO3 and CXCL16 gene contributes to CXCL16 upregulation in CAFs

**Fig. 5:**
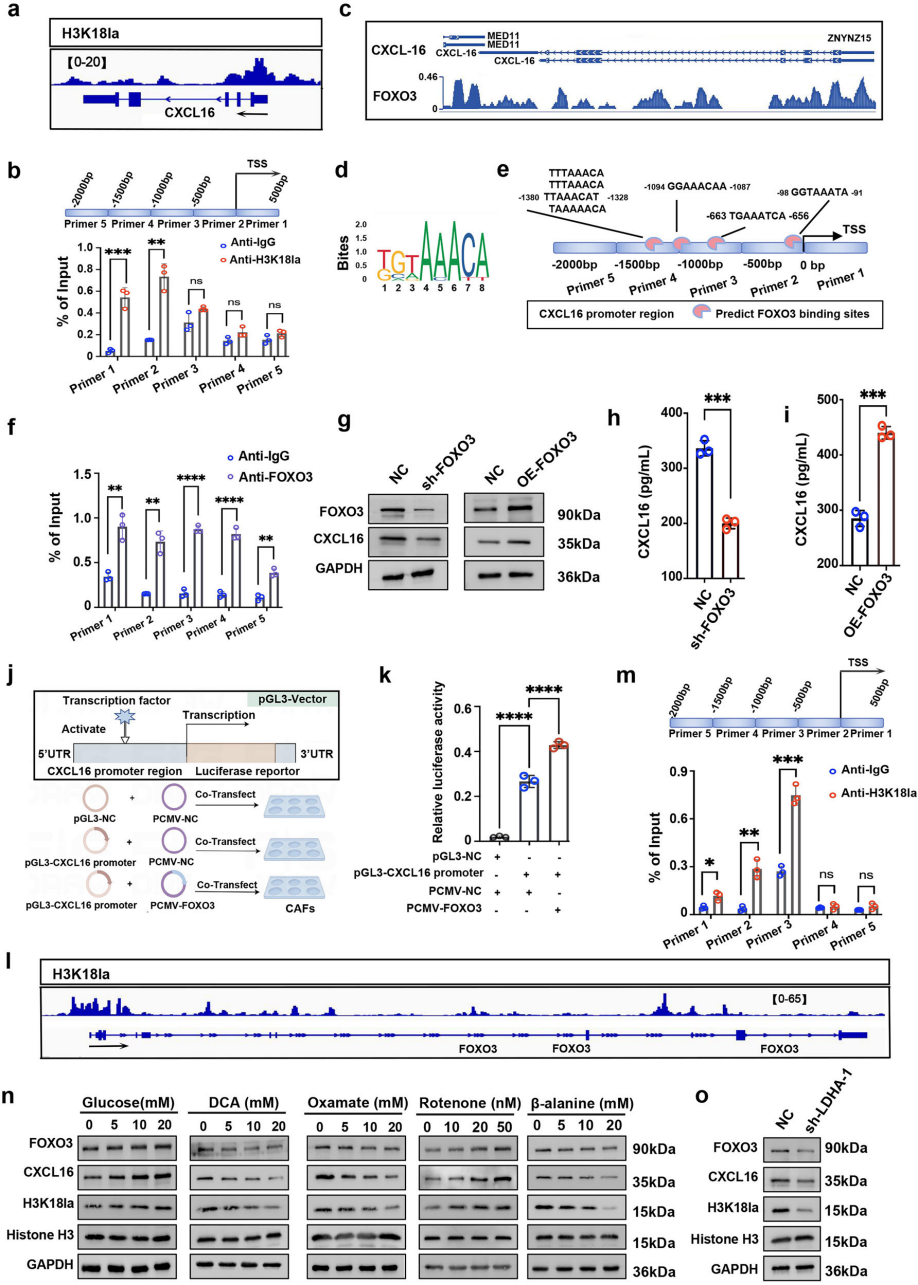
H3K18la enrichment at FOXO3 and CXCL16 promoters upregulates CXCL16 expression in CAFs. **a** IGV tracks presenting H3K18la enrichment at the CXCL16 gene locus in CAFs from MPE by CUT&Tag analysis. **b** ChIP–qPCR validation of H3K18la binding at the CXCL16 promoter. **c**–**e** Predicted FOXO3 binding sites at the CXCL16 promoter using JASPAR (http://jaspar.genereg.net). **f** ChIP–qPCR validation of FOXO3 binding at the CXCL16 gene promoter in CAFs. **g** Western blot analysis of FOXO3 and CXCL16 expression following FOXO3 knockdown or overexpression in CAFs. **h**, **i** CXCL16 levels in CAFs supernatants after FOXO3 knockdown (**h**) or overexpression (**i**). **j** Schematic of dual-luciferase reporter assay, drawn by Figdraw. **k** Luciferase assays assessing FOXO3-mediated regulation of CXCL16 transcription in CAFs. **l**, **m** CUT&Tag (**l**) and ChIP–qPCR (**m**) analyses showing H3K18la enrichment at the FOXO3 promoter. **n** Western blot analysis of the indicated proteins in CAFs treated with glucose (0–20 mm/l), DCA (0–20 mm/l), oxamate (0–20 mm/l), retenone (0–50 nm/l) or β-alanine (0–20 mm/l) for 48 h. **o** Western blot analysis of the indicated proteins in LDHA-knockdown CAFs. Data shown in **b**, **f**–**i**, **k** and **m**–**o** are representative of at least three independent experiments (mean ± s.d.). Statistical analysis was performed using unpaired two-tailed Student’s *t*-test (**h** and **i**) or one-way ANOVA (**b**, **f**, **k** and **m**). *P < 0.05, **P < 0.01, ***P < 0.001, ****P < 0.0001. ns not significant, IGV Integrative Genomics Viewer, OE overexpression.

To elucide how H3K18la modulates CXCL16 expression in CAFs, Cut&Tag analysis was performed using an anti-H3K18la antibody, revealing clear enrichment of H3K18la at the promoter region of CXCL16 gene in CAFs (Fig. 5a). To pinpoint the exact binding sites, we conducted ChIP–qPCR assays using five pairs of primers targeting distinct regions of the CXCL16 promoter, which showed that peak H3K18la levels were located within ±500 bp of the transcriptional start site, particularly in the regions amplified by primer 1 and primer 2 (Fig. 5b). The findings indicate that H3K18la may directly modulate CXCL16 expression by binding its core promoter regions in CAFs.

Using the JASPAR database and Cistrome Data Browser, we predicted FOXO3 as a candidate transcription factor of CXCL16 (Fig. 5c, d), with confirmed the binding sites extending up to 1.5 kb upstream of the CXCL16 promoter by ChIP–qPCR (Fig. 5e, f). Functionally, FOXO3 knockdown in CAFs led to a substantial reduction in CXCL16 expression, while its overexpression increased CXCL16 expression (Fig. 5g–i). Luciferase reporter assay further identified that FOXO3 markedly enhanced the transcription activity of CXCL16 (Fig. 5j, k). Notably, both Cut&Tag analysis and ChIP–qPCR assay consistently revealed substantial H3K18la enrichment in the promoter of FOXO3 in CAFs (Fig. 5l, m), implying that H3K18la may also indirectly enhance CXCL16 transcription by upregulating FOXO3. In line with this, inhibition of glycolysis-induced lactate production through DCA, oxamate treatment or knockdown of LDHA with sh-LDHA-1 (identified as the robust knockdown efficiency in Fig. 4g, h) in CAFs substantially decreased H3K18la levels, as well as FOXO3 and CXCL16 expression (Fig. 5n, o). By contrast, stimulation with glucose and rotenone produced the opposite effects (Fig. 5n).

Taken together, these results suggest that H3K18la in CAFs may promote CXCL16 expression though two distinct mechanism: directly binding to the promotor region and indirectly by increasing FOXO3 expression, which amplifies CXCL16 transcription.

### Targeting lactate production and histone lactylation suppresses MPE progression

**Fig. 6:**
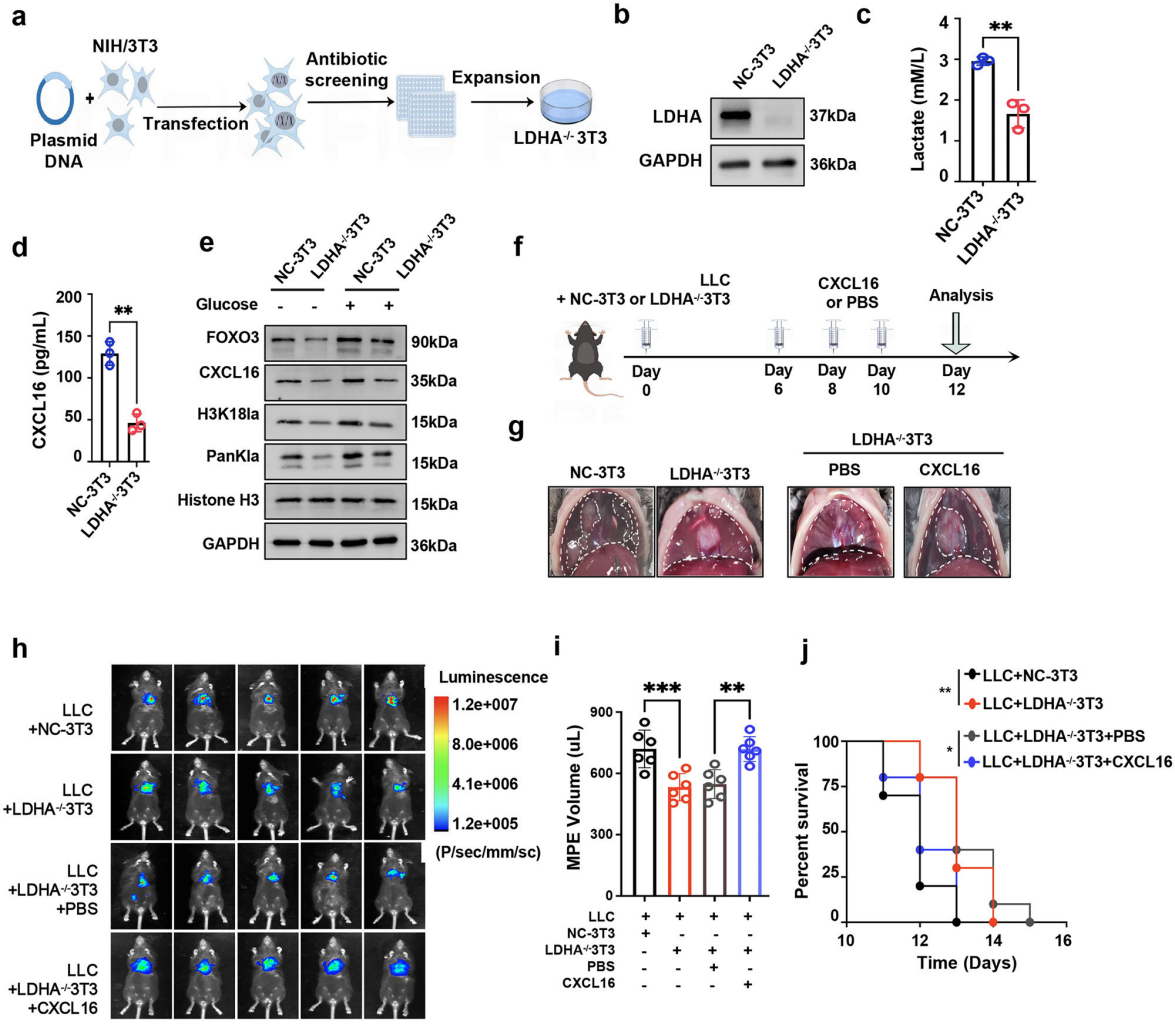
Inhibition of the endogenous lactate production in CAFs suppresses MPE progression. **a** Schematic illustrating the the generation of LDHA-knockout NIH/3T3 fibroblasts (LDHA^−/−^3T3) using CRISPR–Cas9, drawn by Figdraw. **b** Western blot validation of LDHA knockout in LDHA^−/−^ 3T3 fibroblasts. **c**, **d** Lactate (**c**) and CXCL16 (**d**) levels in the culture supernatant of LDHA^−/−^ 3T3 fibroblasts. **e** Western blot analysis of the indicated proteins in LDHA^−/−^ 3T3 fibroblasts cultured with or without glucose. **f** Schematic of MPE mouse model. **g** Representative images showing mouse MPE and thoracic tumors in each group. **h**–**j,** Bioluminescence images depicting the growth (*n* = 5) (**h**), MPE volume (*n* = 6) (**i**) and Kaplan–Meier survival curves (n = 10 per group) (**j**) of MPE-bearing mice. Data shown in **c**–**e** and **g**–**j** are representative of at least three independent experiments (mean ± s.d.). Statistical analysis was performed using unpaired two-tailed Student’s *t*-test (**c** and **d**), one-way ANOVA (**i**) and log-rank test (**j**). *P < 0.05, **P < 0.01, ***P < 0.001, ns not significant, LDHA−/−3T3 LDHA-knockout NIH/3T3 fibroblasts.

To evaluate the impact of CAFs-induced lactate production on MPE development, we further generated an LDHA-knockout NIH/3T3 cell line (Fig. 6a b). For in vitro validation, knockout of LDHA significantly reduced lactate production (Fig. 6c) and led to marked decreases in the levels of FOXO3, CXCL16 and H3K18la, even under glucose-replete conditions (Fig. 6d, e). Functionally, LDHA-deficient fibroblasts displayed impaired ability to attract TNFR2^+^ T_reg_ cells in Transwell assays, which was restored upon addition of exogenous CXCL16 (Supplementary Fig. 8a–c). This highlights the importance of the lactate–H3K18la–CXCL16 axis in TNFR2^+^ T_reg_ recruitment.

**Fig. 7:**
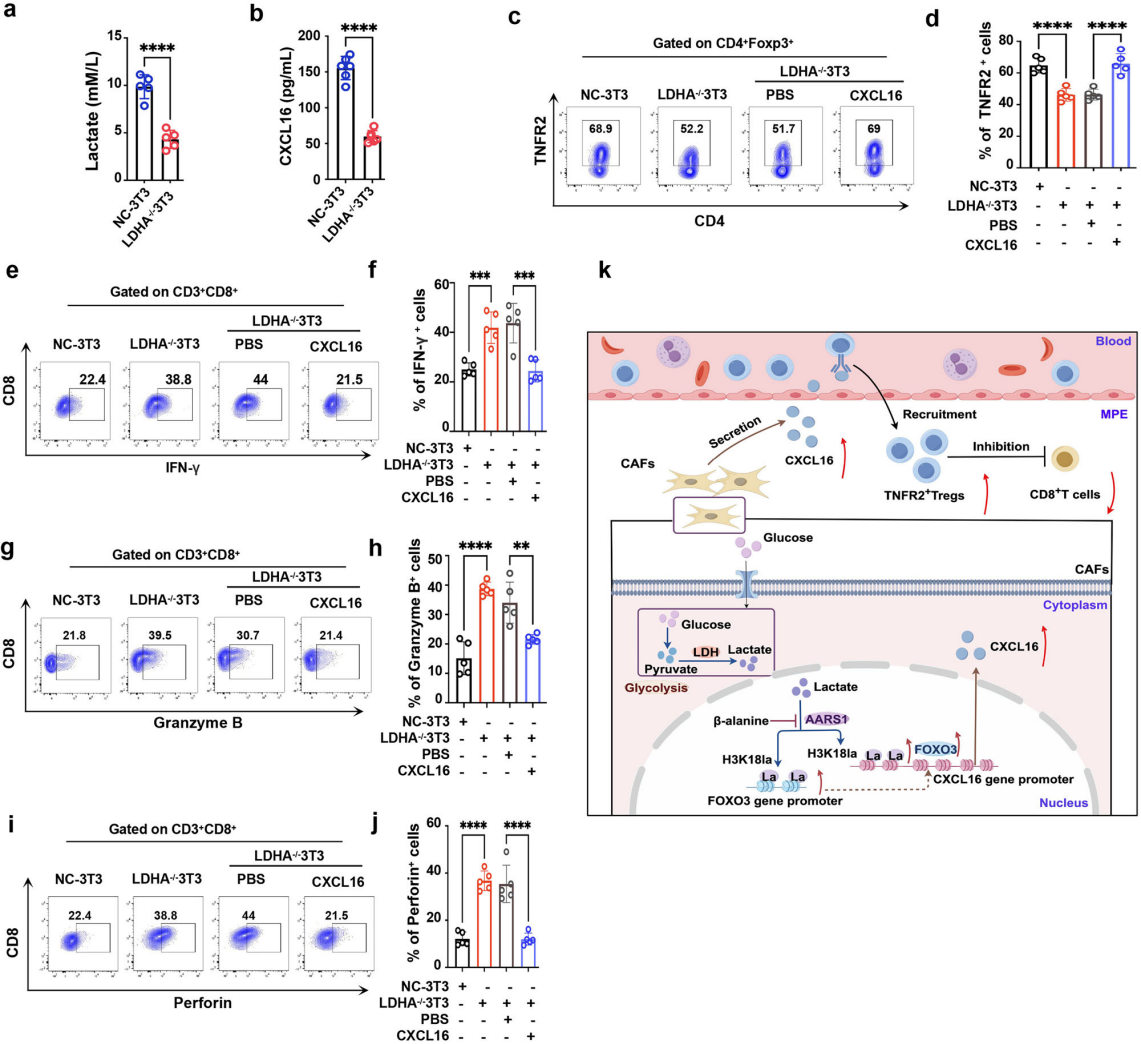
Inhibition of the endogenous lactate production of CAFs alleviates the immunosuppression in MPE. **a**, **b** Lactate (**a**) and CXCL16 (**b**) levels measured in MPE from mice treated with LDHA^−/−^ 3T3 fibroblasts or control fibroblasts. **c**–**j** Flow cytometry plots (**c**, **e**, **g** and **i**) and comparisons (**d**, **f**, **h** and **j**) of the frequencies of TNFR2^+^ T_reg_ cells (**c** and **d**), IFN-γ^+^CD8^+^ T cells (**e** and **f**), granzyme B^+^CD8^+^ T cells (**g** and **h**) and perforin^+^CD8^+^ T cells (**i** and **j**) in MPE. **k** Schematic diagram illustrating the proposed mechanism: CAFs in MPE undergo glycolysis, leading to elevated endogenous lactate levels. This increase in lactate induces H3K18 lactylation modification at the promoter regions of both the CXCL16 gene and its transcription factor FOXO3, thereby promoting CXCL16 expression. TNFR2^+^ T_reg_ cells, which express high levels of CXCR6, the only known receptor for CXCL16, are efficiently recruited into MPE. This recruitment dampens the antitumor response generated by CD8^+^ T cells, leading to the immunosuppression and progression of MPE (by Figdraw). Data shown in **a**–**j** are representative of at least three independent experiments (mean ± s.d.). Statistical analysis was performed using unpaired two-tailed Student’s *t*-test (**a** and **b**) and one-way ANOVA (**d**, **f**, **h** and **j**). **P < 0.01, ***P < 0.001, ****P < 0.0001. ns not significant.

For in vivo validation, LDHA-knockout NIH/3T3 fibroblasts were injected into the pleural cavity of MPE mice (Fig. 6f). Compared with controls, LDHA-knockout fibroblasts significantly reduced the volume of MPE (Fig. 6g–i) and prolonged the survival time of MPE mice (Fig. 6j). Importantly, these effects were reversed by intrapleural CXCL16 administration (Fig. 6g–j). Immune profiling revealed that MPE from mice receiving LDHA-knockout NIH/3T3 fibroblasts showed significantly lower levels of lactate (Fig. 7a) and CXCL16 (Fig. 7b), accompanied by reduced TNFR2^+^ T_reg_ proportions (Fig. 7c, d), and enhanced CD8^+^ T cell cytotoxicity, as indicated by increased IFN-γ (Fig. 7e, f), granzyme B (Fig. 7g, h) and perforin expression (Fig. 7i, j). In addition, the expression of exhaustion markers PD-1 and TIM-3 on CD8^+^ T cells were significantly reduced (Supplementary Fig. 9a–d). These effects were reversed upon CXCL16 reintroduction (Fig. 7c–j and Supplementary Fig. 9a–d), supporting the role of the glycolysis–lactate–CXCL16 axis in mediating the immunosuppression of MPE.

To further strengthen the findings, we assessed pharmacological inhibitors of lactate production and histone lactylation in vivo (Supplementary Fig. 10a, b). Treatment with oxamate or β-alanine significantly reduced MPE burden (Supplementary Fig. 10c), improved survival (Supplementary Fig. 10d) and diminished TNFR2^+^ T_reg_ infiltration in MPE (Supplementary Fig. 10e, f). These interventions strategies recapitulated the effects observed in the LDHA-knockout model, reinforcing the mechanistic link between lactate metabolism, histone lactylation (particularly H3K18la) and MPE progression, while highlighting the therapeutic potential of targeting this metabolic–epigenetic axis.

## Discussion

Our previous work demonstrated that TNFR2^+^ T_reg_ cells accumulate in MPE, exhibiting high levels of inhibitory molecules and potent immunosuppressive functions that accelerate MPE progression^14,15^. In this study, we identified the CXCL16–CXCR6 axis as a crucial mediator for the recruitment of TNFR2⁺ T_reg_ cells to MPE, with CAFs contributing substantially to CXCL16 production. Under hypoxic conditions characteristic of the pleural environment, CAFs exhibit enhanced glycolytic activity, resulting in increased lactate levels. This lactate was associated with elevated H3K18 lactylation at the promoters of CXCL16 and FOXO3, which may stimulate CXCL16 transcription. Subsequently, TNFR2^+^ T_reg_ cells with high levels of CXCR6, the only receptor for CXCL16, could be attracted into MPE, further exacerbating immunosuppression and progression (Fig. 7k).

Despite previous studies reporting enrichment of TNFR2^+^ T_reg_ cells in the tumor microenvironments^9,35–37^, the mechanisms underlying their recruitment remain poorly understood. One study implicated the CCL22–CCR4 axis in TNFR2^+^ T_reg_ accumulation in ovarian cancer ascites^11^. Although we observed similar CCR4 upregulation on TNFR2^+^ T_reg_ cells, the concentrations of CCL22 in MPE displayed marked interpatient heterogeneity with broader expression ranges, suggesting this pathway alone is insufficient for their recruitment. Instead, our data showed the CXCL16–CXCR6 axis as the dominant pathway, given the consistent elevation of CXCL16 in MPE and selective co-expression of CXCR6 with TNFR2 on T_reg_ cells. Furthermore, TCGA survival analysis revealed that high CXCL16 expression correlated with poor prognosis and showed positive associations with both Foxp3 and TNFR2 across multiple malignancies, underscoring the broader relevance of the CXCL16–CXCR6 axis in TNFR2^+^ T_reg_ recruitment.

In the current study, we identified CAFs as the main drivers of the CXCL16–CXCR6 axis in MPE, attributed to their elevated CXCL16 expression, aligning with the well-known secretory characteristics of CAFs in tumor environment^18^. Although our data indicate CAFs as the major source of CXCL16 in MPE, we acknowledge that neutrophils and macrophages also express CXCL16, consistent with previous reports^38,39^. Given the well-established roles of tumor-associated macrophages and tumor-associated neutrophils in maintaining immunosuppressive microenvironment via high expression of immune checkpoint molecules and cytokines^25,40^, it is plausible that CXCL16-mediatd TNFR2^+^ T_reg_ recruitment may represent an additional mechanism by which these myeloid cells amplify immunosuppression. Future studies dissecting the the contributions of CXCL16-producing neutrophils and macrophages may provide a more comprehensive understanding of the immunosuppressive landscape in MPE.

Under hypoxic conditions, CAFs exhibited a time-dependent increase in CXCL16 expression. Due to the sealed pleural cavity^41^, local hypoxia as a critical characteristic of MPE, providing a metabolic context conducive to CXCL16 upregulation in CAFs. Lactate, a byproduct of hypoxia-induced glycolysis, has emerged as a regulator of lactylation, a novel epigenetic modifications^21–23^. Among various lactylation sites, lactylation at H3K18 appears important for transcriptional activation^21,30,31^. In cisplatin-resistant bladder cancer cells, H3K18la upregulates YBX1 and YY1 expression to confer cisplatin resistance^42^. Similarly, in pancreatic ductal adenocarcinoma, H3K18la enrichment regulates mitotic checkpoint regulators TTK and BUB1B transcription to tumorigenesis^43^. In line with these findings, our data suggest that CAFs-derived lactate induces H3K18la at the promoter regions of both the CXCL16 gene and its transcription factor FOXO3, which may stimulate CXCL16 expression. This subsequently attracts TNFR2^+^ T_reg_ cells into MPE, promoting the immunosuppression and progression of MPE.

Clinical evidence further supports these findings. Patients with higher levels of H3K18la in CAFs exhibit elevated concentrations of CXCL16 and an increased proportion of TNFR2^+^ T_reg_ cells in MPE. Consistently, elevated H3K18la levels in CAFs correlated positively with greater tumor burden, larger MPE volume and worse performance status, further highlighting their involvement in MPE progression. Moreover, CUT&Tag analysis identified multiple target genes of H3K18la in CAFs beyond CXCL16 and FOXO3. Further studies are warranted to elucidate the functions of these targets and the broader role of H3K18la in modulating CAF-driven immunoregulation in MPE.

Initially, the acetyltransferase p300 was proposed as a lactyltransferase mediating histone lactylation. However, the low concentration of its proposed lactyl donor, lactyl-coenzyme A, has raised doubts about its role as a genuine lactyltransferase^33^. More recently, AARS1 has been proposed as a more plausible lactyltransferase, as it directly utilizes lactate and ATP to catalyze protein lactylation^32^. Consequently, β-alanine, which disrupts lactate binding to AARS1, has been used as an inhibitor of lactyltransferase activity^32^. In our current study, we observed a substantial reduction in H3K18la levels in CAFs following β-alanine treatment, suggesting that β-alanine effectively inhibited AARS1-mediated lactylation processes. Nonetheless, future research should focus on developing more specific inhibitors that target AARS1 to refine therapeutic strategies aimed at lactylation pathways.

Despite the novel insights provided by our study, several technical limitations hindering the precise investigation of H3K18 lactylation should be acknowledged. Specific inhibitors or genetic tools capable of selectively targeting H3K18la are still unavailable, making it difficult to perform definitive loss-of-function studies. In addition, generating histone point mutation models in primary CAFs or in vivo remains technically challenging due to low transfection efficiency and cellular heterogeneity. Although LDHA knockout, oxamate and β-alanine treatment indirectly modulate histone lactylation and produced consistent changes in H3K18la and CXCL16 expression, these approaches cannot fully isolate the specific function of H3K18la and may introduce off-target effects. These limitations highlight the urgent need for more precise tools to directly interrogate the biological role of H3K18la in cancer progression.

Nevertheless, this work provides evidence implicating histone lactylation in CAFs as an epigenetic regulatory mechanism underlying MPE immunosuppression, offering insights for therapeutic development.

## Supplementary information

Supplementary Information

## Data Availability

The datasets used during the current study are available from the corresponding author on reasonable request. The raw data generated in this study are available via the Sequence Read Archive at https://www.ncbi.nlm.nih.gov/sra/ with BioProject accession no. PRJNA1170877. The single-cell RNA-seq datasets from MPE^25^ are available from SRA: PRJNA970083.
