# Supplementary material for: H3K18 lactylation in cancer-associated fibroblasts drives malignant pleural effusion progression via TNFR2+ Treg recruitment
Source: Exp Mol Med. 2025 Nov 28;57(11):2671–85. doi: 10.1038/s12276-025-01557-3 (PMC12686498; doi:10.1038/s12276-025-01557-3)
Supplement: Supplementary file 1 — Supplementary Information [file 12276_2025_1557_MOESM1_ESM.pdf]

Supplementary Information

H3K18 Lactylation in Cancer-Associated Fibroblasts Drives Malignant Pleural Effusion Progression via TNFR2⁺ Tregs Recruitment

Linlin Ye[1*], Xuan Xiang[1*], Zihao Wang[1*], Siyu Zhang[1,2*], Qianqian Xue[1], Xiaoshan Wei[1,3], Yao Liu[1], Haolei Wang[1], Jiaqi Ai[1], Bohan Yang[1], Long Chen[1], Yiran Niu[1,4], Wenbei Peng[1#], Qiong Zhou[1#]

1 Department of Respiratory and Critical Care Medicine, Union Hospital, Tongji Medical College, Huazhong University of Science and Technology, Wuhan, 430022, China

2 Department of Respiratory and Critical Care Medicine, Tongji Hospital, Tongji Medical College, Huazhong University of Science and Technology, Wuhan, Hubei, 430030, China

3 Department of Respiratory and Critical Care Medicine, The First Affiliated Hospital of Guangxi Medical University, Nanning, Guangxi, China

4 Department of Respiratory and Critical Care Medicine, The Central Hospital of Wuhan, Tongji Medical College, Huazhong University of Science and Technology, Wuhan, China.

* These authors contributed equally to this work.

[#] **Corresponding authors:**

Qiong Zhou. E-mail: zhouqiongtj@hust.edu.cn

Department of Respiratory and Critical Care Medicine, Union Hospital, Tongji Medical College, Huazhong University of Science and Technology, Jiefang Avenue 1277, Wuhan, China. 430022.

Wenbei Peng. E-mail: pengwenbei_@hust.edu.cn

Department of Respiratory and Critical Care Medicine, Union Hospital, Tongji Medical College, Huazhong University of Science and Technology, Wuhan, China. 430022.

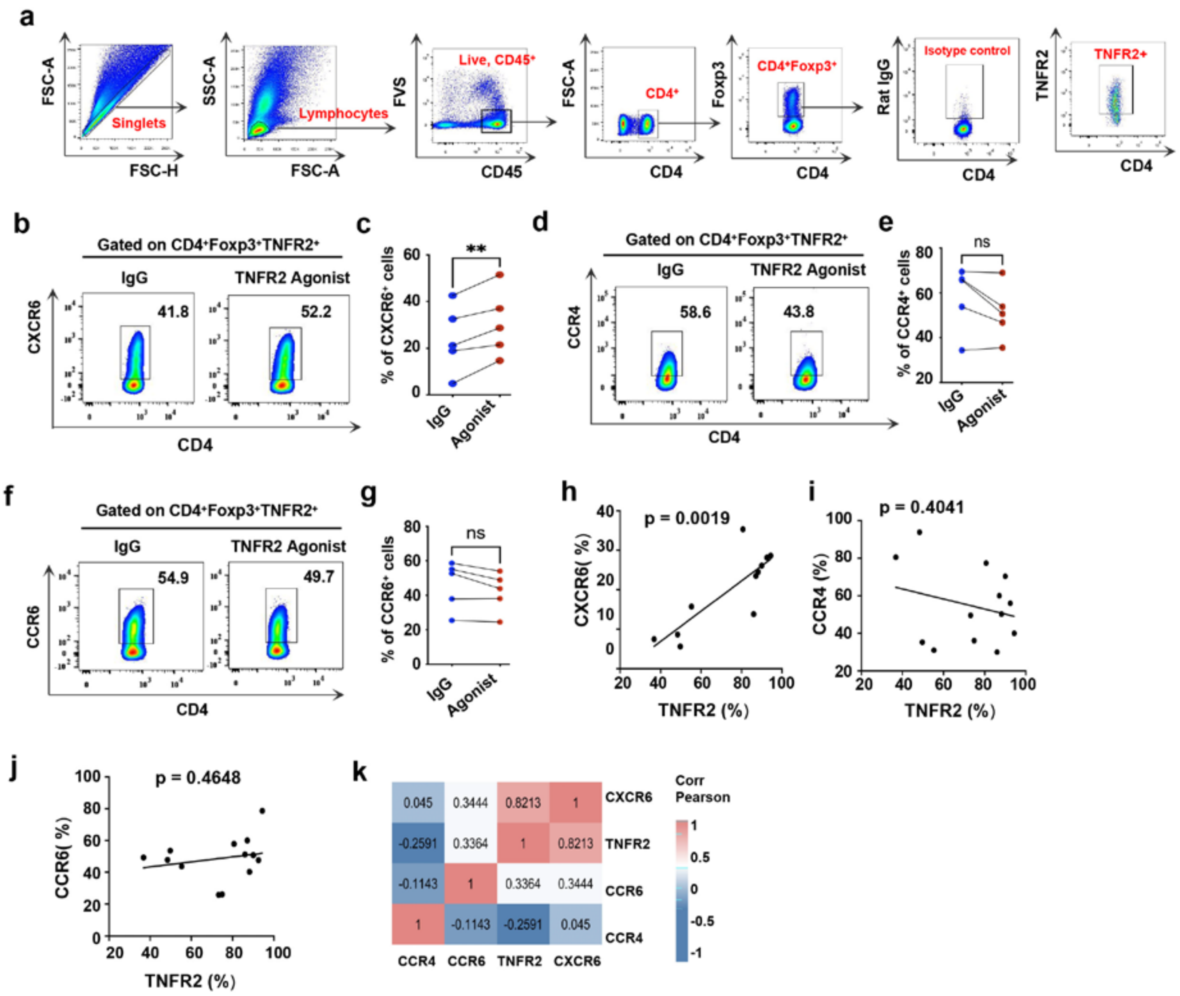

Supplementary Fig. 1 CXCR6 and TNFR2 co-expressed by Tregs in MPE.

(a) Flow cytometry gating strategies for human TNFR2^{+}Tregs. (b-g) CD4^{+}T cells purified from human MPE were treated with TNFR2 agonist (2.5 μg/mL) or its isotype control for 48 hours. Flow cytometry analysis of (b-c) CXCR6, (d-e) CCR4 and (f-g) CCR6 expression on TNFR2^{+}Tregs at the indicated conditions (n=5). Correlation between TNFR2 expression and (h) CXCR6, (i) CCR4, (j) CCR6 levels on Tregs (n=13). (k) Correlation heatmap illustrating the relationships among CXCR6, CCR4, CCR6 and TNFR2 expression on Tregs. Data are representative of at least three independent experiments. Statistical analysis was performed using paired two-tailed Student's t-test (c, e, g), and Correlations

were determined by Pearson's rank correlation coefficients (h-j). **p<0.01, ns, not significant. FVS, Fixable Viability Stain 780.

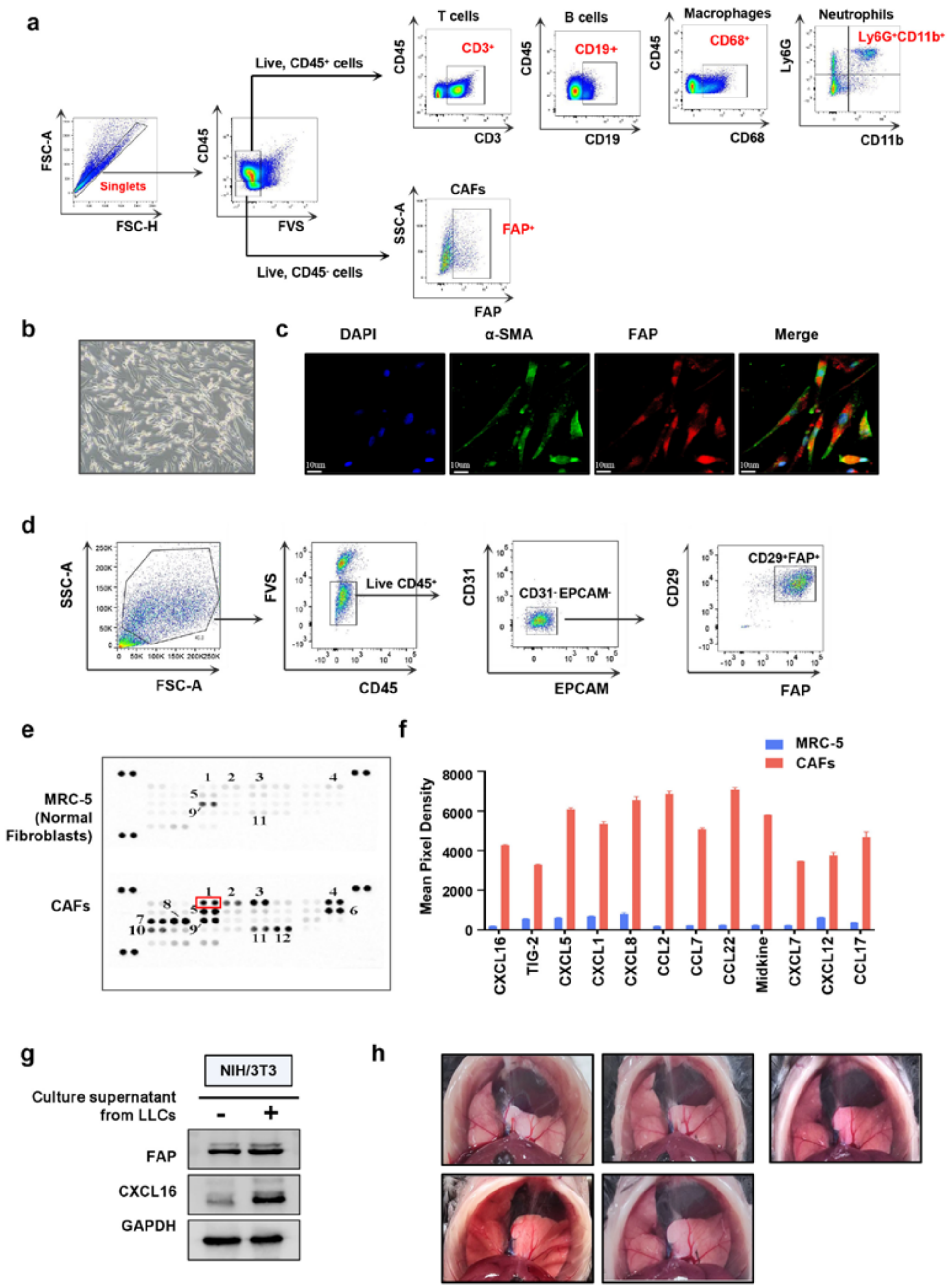

Supplementary Fig. 2 Related to Figure 2.

(a) Flow cytometry gating strategies for human CAFs, T cells, B cells, macrophages and neutrophils in MPE. (b) Microscope image of CAFs purified from human MPE using MACS. (c) Immunofluorescence

images of α-SMA and FAP staining in CAFs. scale bar 10 μm. (d) Flow cytometry analysis of CAF purity following MACS isolation. (e-f) Comparisons of chemokines expression between MPE-derived CAFs and normal human lung fibroblasts (MRC-5) using a Human Chemokine Array Kitand (f) and quantify expression density via ImageJ software (f). (g)Western blot analysis of FAP and CXCL16 expression in NIH/3T3 fibroblasts (normal mouse fibroblast cell line) treated with culture supernatant from LLCs for five days. (h) Representative images of the pleural cavity 14 days after the injection of NIH/3T3 fibroblasts alone (n=5).MACS, magnetic-activated cell sorting; FAP,fibroblast activation protein; α-SMA, alpha-smooth muscle actin; EpCAM, Epithelial Cell Adhesion Molecule; LLCs, murine Lewis lung carcinoma cells.

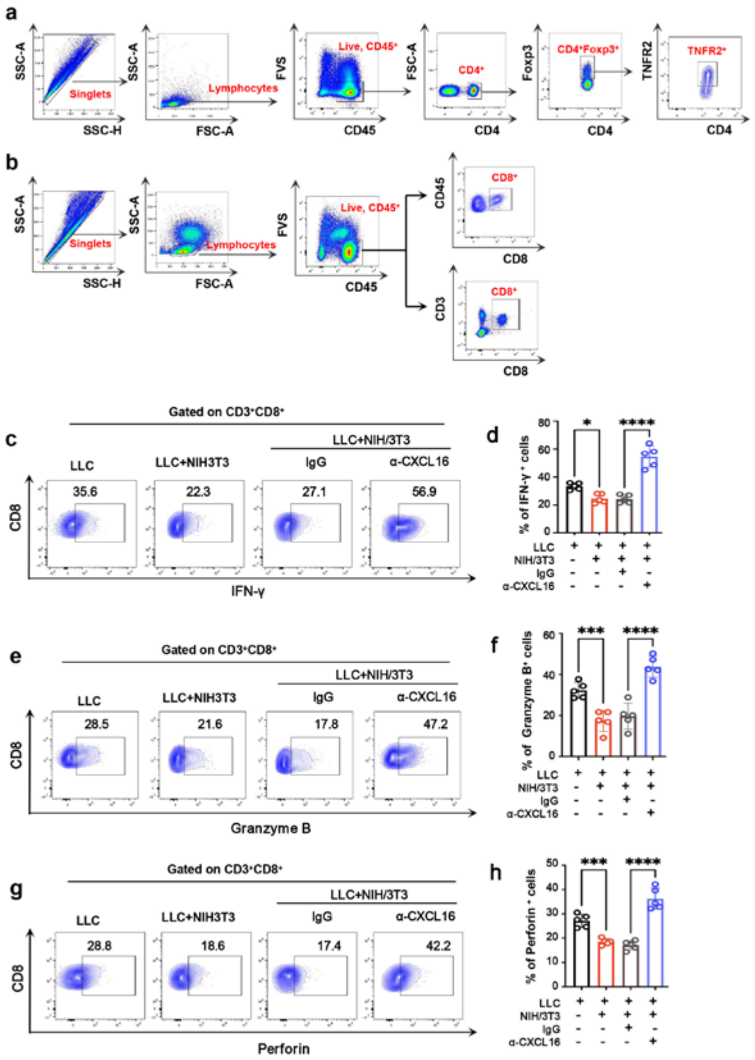

Supplementary Fig. 3 CAF-derived CXCL16 impairs the cytotoxicity of CD8$^+$T cells in MPE. (a-b) Flow cytometry gating strategies for mouse TNFR2$^+$Tregs and CD8$^+$Tcells. (c-h) Flow cytometry analysis of the frequencies of (c-d) IFN-γ$^+$cells, (e-f) granzyme B$^+$cells and (g-h) perforin$^+$cells in CD8$^+$T cells from murine MPE within each groups. Data shown in (c-h) are representative of at least three independent experiments (mean±SD). Statistical analysis was performed using one-way ANOVA (D, F, H). *p<0.05, ***p<0.001, ****p<0.0001. ns, not significant; α-CXCL16, CXCL16 neutralizing antibody.

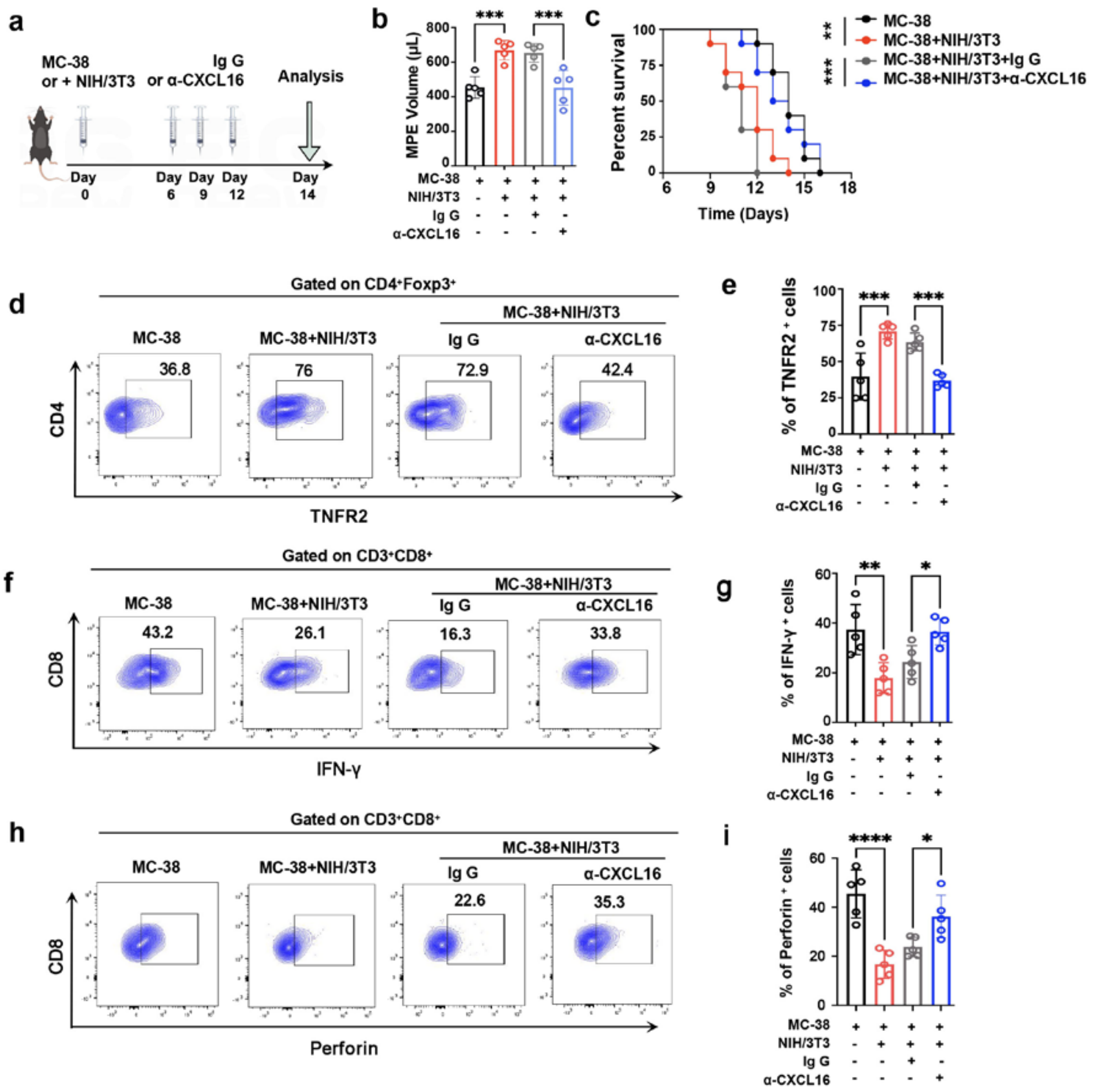

Supplementary Fig. 4 CAF-derived CXCL16 promotes TNFR2$^+$Tregs recruitment and disease progression in MC38 -induced MPE models.

(a)Schematic diagram of the MC38-induced MPE model. (b) MPE volume (n=5) and (c) Kaplan-Meier survival curves in each group (n=10 per group). (d-e) Frequencies of TNFR2$^+$Tregs in MC38-induced MPE within each group. (f-i) Proportions of (f-g) IFN-γ$^+$and (h-i) perforin$^+$cells in CD8$^+$T cells from murine MPE within each groups. Data shown in (b-i) are representative of at least three independent experiments (mean±SD). Statistical analysis was performed using one-way ANOVA (b,e,g and i) or

log-rank test (c). *p<0.05, **p<0.01, ***p<0.001,****p<0.0001. ns, not significant. MC38 cells, murine colon cancer cells.

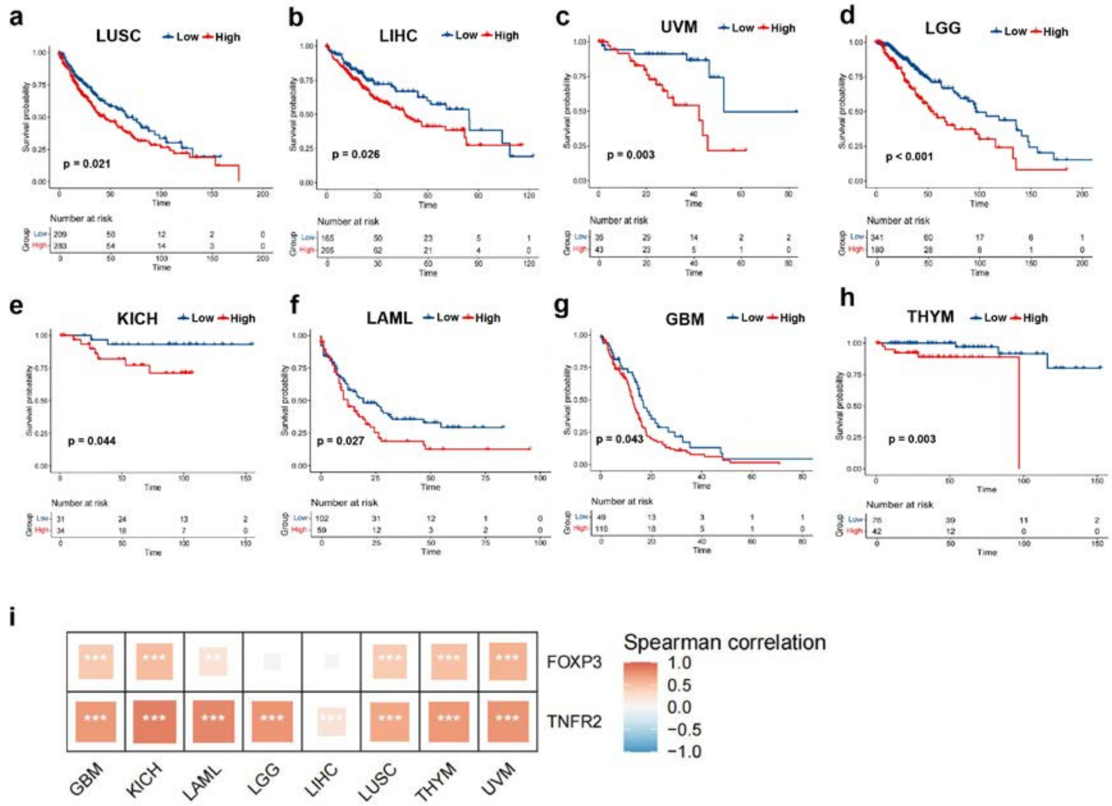

Supplementary Fig. 5 High CXCL16 expression correlates with poor prognosis across multiple cancer types.

(a-h) Kaplan-Meier survival curves comparing overall survival (OS) between CXCL16 ^{high} and CXCL16^{low} expression groups in patients with (a) lung squamous cell carcinoma (LUSC), (b) liver hepatocellular carcinoma (LIHC), (c) uveal melanoma (UVM), (d) lower-grade glioma (LGG), (e) kidney chromophobe (KICH), (f) acute myeloid leukemia (LAML), (g) glioblastoma multiforme (GBM), and (h) thymoma (THYM), based on The Cancer Genome Atlas (TCGA) datasets (i) Heatmap-style dot plot illustrating the correlations between CXCL16, TNFR2 and Foxp3 expression across diverse cancer

types. Statistical analysis was performed using log-rank test (a-h) and Spearman's rank correlation coefficients (i).***p < 0.001.

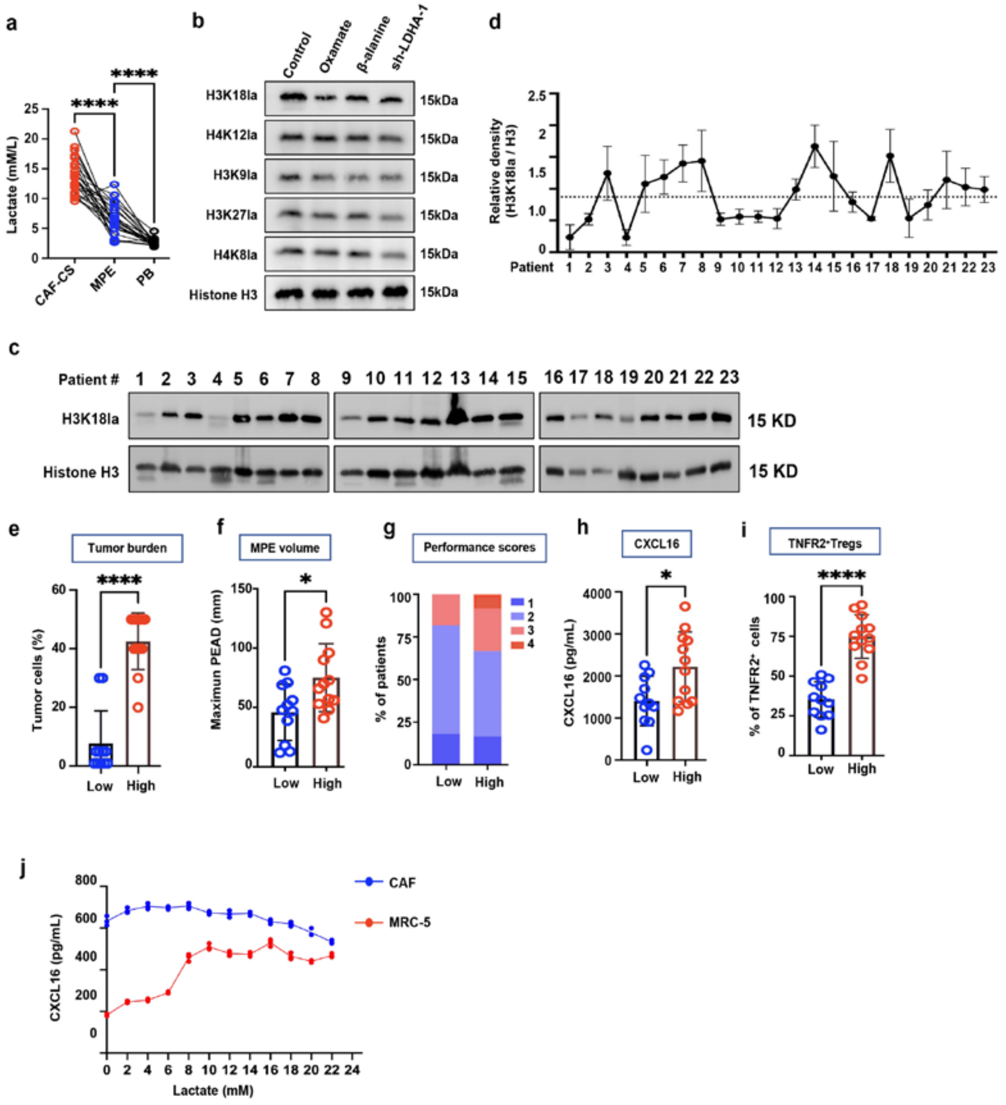

Supplementary Fig. 6 H3K18 lactylation in CAFs as a predictor of poor prognosis of MPE patients.

Twenty-three MPE specimens and the corresponding peripheral blood of patients with untreated lung cancer were collected. CAFs were isolated from MPE using MACS. The purified CAFs were cultured in vitro for five days, and the culture supernatant were collected. (a) Lactate levels in CAFs supernatants

(CAF-CS), MPE and PB (n = 23). (b)Western blot analysis of the histone lactylation levels at different sites in CAFs or treated with Oxamate (20mm/L) or β-alanine (20mm/L) for 48 hours, and in LDHA-konckdown CAFs. (c-d) H3K18la levels in CAFs detected using Western blot and quantified by Image J software (n = 23). Based on H3K18la levels in CAFs, patients were stratified into H3K18la^{high} (n = 12) and H3K18la^{low} (n = 11) groups. Comparisons between the two groups: (e) tumor cell proportions in MPE (from pathological report), (f) maximum pleural effusion anteroposterior diameter (PEAD, measured by ultrasonography), (g) distribution of ECOG PS scores, (h) CXCL16 levels in MPE, and (i) frequencies of TNFR2^{+} Tregs in MPE. (j) CAFs and MRC-5 (normal human lung fibroblasts) were treated with exogenous lactate (0-22mM) for 48 hours, and CXCL16 levels in the culture supernatant were measured by ELISA. Data are representative of at least three independent experiments (mean±SD). Statistical analysis was performed using one-way ANOVA(A) or unpaired two-tailed Student’s t-test (E-F, H-I). *$p<0.05$, ****$p<0.0001$. CS, culture supernatant; H3K18la, lactylation at histone H3 lysine 18; ECOG PS scores, Eastern Cooperative Oncology Group Performance Status scores.

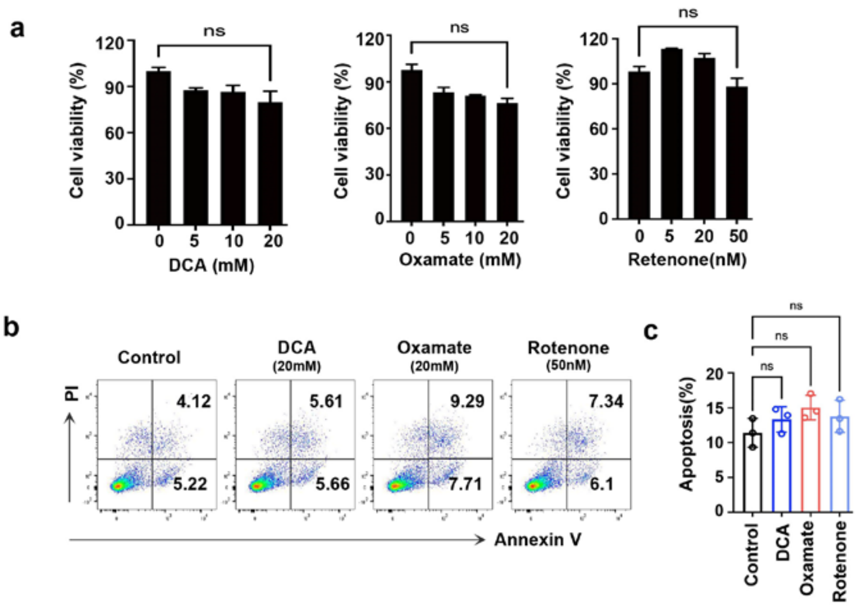

Supplementary Fig. 7 Effects of metabolic modulators on CAFs viability and apoptosis.

CAFs were treated with DCA (0-20mM), Oxamate (0-20mM) or Retenone (0-50nM)for 48 hours. (a)Cell viability was assessed using CCK-8 assay. (b-c) Flow cytometry analysis of apoptosis with Annexin V-FITC/PI staining in CAFs after 48-hour treated with DCA (20mM), Oxamate (20mM) or Retenone (50nM). Data are representative of at least three independent experiments. Statistical analysis was performed using Mann-Whitney test (a) or one-way ANOVA(c).ns, not significant. PI, propidium iodide; CCK-8, Cell Counting Kit-8

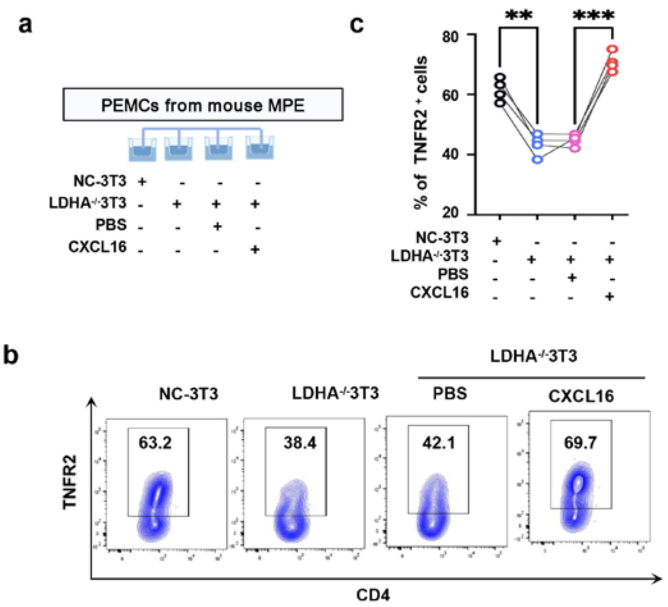

Supplementary Fig. 8 LDHA knockout in fibroblasts reduces TNFR2^{+}Tregs recrutment in vitro

(a) Schematic diagram of the chemotaxis assay testing TNFR2^{+}Tres recruitment under the specified conditions (By Figdraw). (b-c) Flow cytometry analysis of TNFR2^{+}Tregs frequencies recruited into the designated media (n =4). Data shown in (b-c) are representative of at least three independent experiments. Statistical analysis was performed using one-way ANOVA. **p<0.01, ***p<0.001.

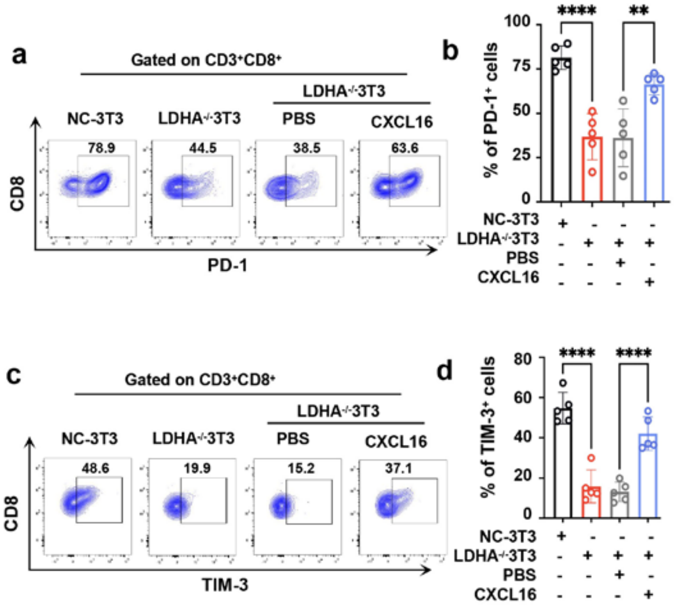

Supplementary Fig. 9. Expression of exhaustion markers on CD8^{+}T cells in murine MPE

Flow cytometry plots and quantification of the frequencies of (a-b) PD-1^{+}cells and (c-d) TIM-3^{+}cells among CD8^{+}T cells in murine MPE across the indicated groups. Data are representative of at least three independent experiments (mean±SD). Statistical analysis was performed using one-way ANOVA (B and D). **p<0.01, ****p<0.0001.

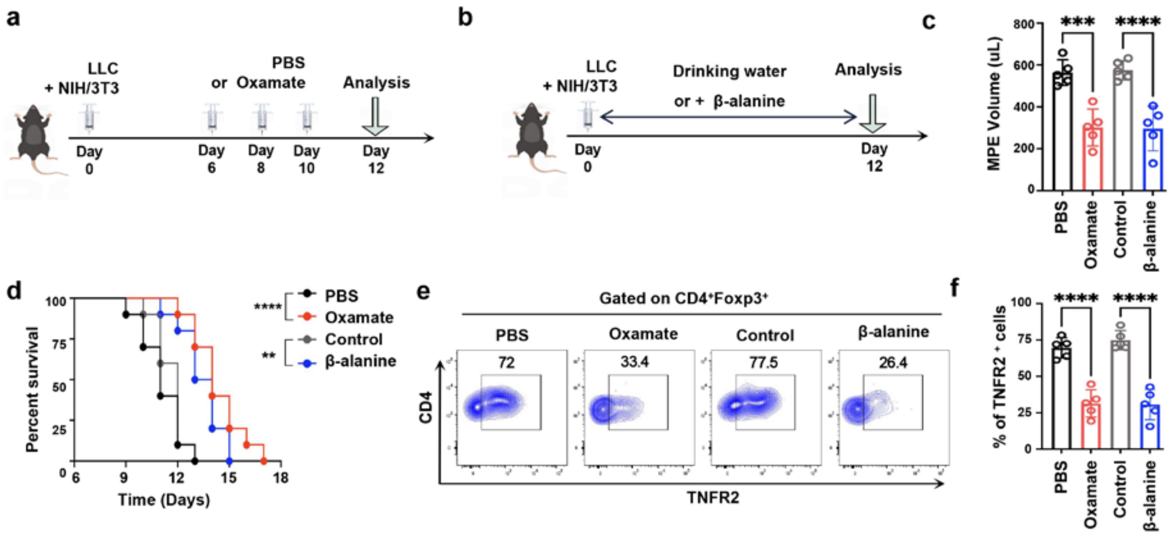

Supplementary Fig. 10. Targeting histone lactylation by oxamate and β-alanine alleviates MPE progression.

(a-b) Schematic illustrating the MPE mouse model and treatment strategy (By FigDraw). (c) MPE volume (n = 5), (d) Kaplan–Meier survival curves and (e-f) frequencies of TNFR2^{+}Tregs in MPE in each treated group. Data shown in (c-f) are representative of at least three independent experiments (mean±SD). Statistical analysis was performed using one-way ANOVA (c and f) or log-rank test (d). **p<0.01, ***p<0.001, ****p<0.0001.

Supplementary Table 1. Detail information of antibodies used in this study.

Antibodies	Clone	Catalogue number	RRID	Manufacturer
**Human antibodies for flow cytometry**				
Brilliant Violet 510™ anti-human CD45 Antibody	HI30	304036	AB_2561940	Biolegend
Percp-cy5.5 anti-human CD3	OKT3	317336	AB_2561628	Biolegend
FITC anti-human CD4	RPA-T4	555346	AB_395751	BD Biosciences
FITC anti-human CD8a	RPA-T8	555366	AB_395769	BD Biosciences
PE-Cy™7 mouse anti-human CD25	M-A251	557741	AB_396847	BD Biosciences
Brilliant Violet 421™ anti-human FOXP3	206D	320124	AB_2565972	Biolegend
PE anti-human CD120b	MR2-1	TNFR7504	AB_10373551	eBiosciences
Abti-hFAP Alexa Flour 700 Conjugated	427819	FAB3715N	AB_3649604	R&D Systems
Percp-cy5.5 anti-human CD325 (EPCAM)	CO17-1A	369803	AB_2650899	Biolegend
PE anti-human CD29	TS2/16	303003	AB_314319	Biolegend
PE-Cyanine7 anti-human CD31	WM59	303118	AB_2247932	Biolegend

PE anti-human CXCL16	22-19-12	360804	AB_2563011	Biolegend
PE/Cy7 anti-human CA11b	ICRF44	301322	AB_830644	Biolegend
PE-Cyanine7 anti-human CD14	63D3	367112	AB_2566714	Biolegend
FITC anti-human CD14	61D3	11-0149-42	AB_10597597	eBiosciences
APC anti-human CD19	SJ25C1	17-0198-42	AB_1272030	eBiosciences
APC anti-human CD186 (CXCR6)	K041E5	356006	AB_2562223	Biolegend
PE/Cyanine7 anti-human CD194 (CCR4)	L291H4	359410	AB_2562431	Biolegend
APC anti-human CD196 (CCR6)	G034E3	353416	AB_10915987	Biolegend
**Mouse antibodies for flow cytometry**				
Brilliant Violet 510™ anti-mouse CD45 Antibody	S18009F	157219	AB_2563061	Biolegend
PerCP/Cyanine5.5 Anti-mouse CD3e	145-2C11	100328	AB_893318	Biolegend
FITC Anti-mouse CD4	RM4-5	100510	AB_312713	Biolegend
FITC Anti-mouse CD8a	53-6.7	100706	AB_312745	Biolegend
Brilliant Violet 605™ anti-mouse CD8a	53-6.7	100744	AB_2562609	Biolegend

Brilliant Violet 421™ anti-mouse FOXP3	MF-14	126419	AB_2565933	Biolegend
Alexa Fluor 647 anti-mouse FOXP3	MF-14	126408	AB_1089115	Biolegend
PE anti-mouse CD120b (TNFR Type II/p75)	TR75-89	113406	AB_2206941	Biolegend
PE anti-human/mouse Granzyme B	QA16A02	372208	AB_2687032	Biolegend
PE/Cyanine7 anti-mouse IFN-γ	XMG1.2	505826	AB_2295770	Biolegend
APC anti-mouse Perforin	S16009A	154304	AB_2721463	Biolegend
PE/Cyanine7 anti-mouse CD279 (PD-1)	RMP1-30	109110	AB_572017	Biolegend
APC anti-mouse CD366 (Tim-3)	RMT3-23	119706	AB_2561656	Biolegend
BD Horizon™ Fixable Viability Stain 780		565388	AB_2869673	BD Biosciences

Supplementary Table 2. Flow cytometry panels.

Cell Subsets	Fluorescence									
	APC-Cy7	FITC	PE	Percy-cy5.5	PE-cy7	APC	BV421	BV510	Alexa Flour 700	BV605
Human-TNFR2^{+}Treg-1	FVS	CD4	TNFR2				Foxp3	CD45		
Human-TNFR2^{+}Treg-2	FVS	CD4	TNFR2		CCR4	CXCR6	Foxp3	CD45		
Human-TNFR2^{+}Treg-3	FVS	CD4	TNFR2			CCR6	Foxp3	CD45		
Human-CAF-1	FVS		CD29	EPCAM	CD31			CD45	FAP	
Human-CAF-2	FVS							CD45	FAP	
Human-Macrograph	FVS				CD68			CD45		
Human-Neutrophils	FVS				CD11b	Ly6G		CD45		
Human-T cells	FVS			CD3				CD45		
Human-B cells	FVS		CD19			CD19		CD45		

Mouse-TNFR2$^+$Treg	FVS	CD4	TNFR2			Foxp3	CD45	
Mouse-CD8$^+$T cells-1	FVS		Granzyme B	CD3	IFN-γ	Perforin	CD45	CD8
Mouse-CD8$^+$T cells-2	FVS	CD8		CD3	PD-1	TIM-3	CD45	

Supplementary Table 3. Sequences of sgRNA in this study.

**Name**	**Organism**	**Sequence** (5'-3')	**Description**
sg-LDHA	Mouse	AGTATCTTAATGAAGGTAAGTGG	CRISPR/Cas9
sg-LDHA	Mouse	CTGCTGATCGTCTCCAATCCAGG	CRISPR/Cas9
sg-LDHA	Mouse	CGGGGGCCCGTCAGCAAGAGGGG	CRISPR/Cas9

Supplementary Table 4. Primer sequences for ChIP-qPCR in this study

**Gene name**	**Sequence** (5'-3')	**Description**
Human FOXO3 promoter	Forward primer: GCGCGAGAGGAGAGCG	CHIP-qPCR primer1
	Reverse primer: GGAAGCCGGTGCCTCTG	
Human FOXO3 promoter	Forward primer: CCTGCGTGTGTCTATAACTTTGTGC	CHIP-qPCR primer2
	Reverse primer: CCACATTCCTCCTCCTCCCTTC	
Human FOXO3 promoter	Forward primer: TTTCCACACCGCGATAACCCT	CHIP-qPCR primer3
	Reverse primer: TGTCCGGTTCCCTGTTAGAACG	
Human FOXO3 promoter	Forward primer: TGGGCTGGACCTTTTTGTGT	CHIP-qPCR primer4
	Reverse primer: TCTACGAATGCACCACACGC	
Human FOXO3 promoter	Forward primer: CGAGTGCGCTGCAACTTTC	CHIP-qPCR primer5
	Reverse primer: CGCACTAGAAACCAATCCTTCG	
Human CXCL16 promoter	Forward primer: GGAAGTGAAAGTGCCTCGGA	CHIP-qPCR primer1
	Reverse primer: GTTCGGTTCAGGAGGCCG	

Human CXCL16 promoter	Forward primer: TCCGTGGATATGCTAGGAGC	CHIP-qPCR primer2
	Reverse primer: AGCCCCCATTTTCCTCCCTA	
Human CXCL16 promoter	Forward primer: AACTCCATGTCTTCAGCGGCA	CHIP-qPCR primer3
	Reverse primer: TGCTCTCCTGTCCCTCACCATA	
Human CXCL16 promoter	Forward primer: TGAAGGCCAGCACTCAGAACTA	CHIP-qPCR primer4
	Reverse primer: GGACAGGGTGGAGAACGAAACA	
Human CXCL16 promoter	Forward primer: CCATGCTCATCCGTCACAAGT	CHIP-qPCR primer5
	Reverse primer: TGACCACATTTGTCTCTCGCTG	

Supplementary Table 5. Oligonucleotides used for luciferase reporter gene.

Oligonucleotides name	Sequence (5'-3')
CXCL16 promoter	ggacagctgctacagaggaccagaatgcgctgccatgcagaaatgtaggcccactgtcagagcttgagatttttcttgggaatgtgtaaatccag<br>acttctggatactttttttcttgcaatgtttaaacattaaattaaaaaaaaaaaccccactgtgggtcaataaaaacaccttttcaggccagattggcct<br>gaaggccagcactcagaactagtggcaggggagggcttccgaagggaggatggctgggcaaggggtagggcagggccagccctgggttct<br>taccactcctctgtcccttcctcttctggggtcttgtttcgttctccaccctgtcctctcggcagccatccttgccacctcctgcctcccgggcagcctc<br>ctctgactctcctggagggtttgtttcctggggggactccctgctggtaggggctagctcccggtcctcctctggttccaccttctctgtgctgcctg<br>cacccccgtcctctctcttctcttcttcttcatcttcctcctcttcctcctcccttcctccccatcctctaggctgacaaagctgatgtaggggctcagg<br>tctcgcaggtggagtggagggttgtctcccaggagccaggcagtccccaggcctggtccaggacctggttctgccccttgccccaggctgatg<br>cccacactatggttgggcaggacatggagcacccaaagggcagggtcctggggtagccttctgatctgggcctccagctgccggcagcggcc<br>ctcaaggctagtccctacagctccacgctctgccacaaactttcggaaccagccgaagagaagggcagtgaaatcaaggaactcatcccggtat<br>ccagacacaaactccatgtcttcagcggcacagggccccggcccagactgagcaaaagaagggatatatggtgagggacaggagagcaatg<br>atggagtttggggctgggtcacaaattgagggagagggtaggtttgagagtcacttagggttggatccgtggatatgctaggagcggagaaga<br>ggatttttcagggctgaggatgggagtttagggaggaaaatgggggctggacttgaaagtgggtgaccagggttggagaagactactcaggga

	tgagatggtatttggggcttaaaattggagagttcacaccctcaaaagcagagtttaaattaaggctagagaggagagagggctaatcctatcgg
	gaacggtttggtggggagggaagcattttggatgcgtctgtggggtatgggagtgggagcgagggggttccctgggctcggatctgcgtggcg
	ggcagccccggtagacctagcccctcccggccgccccactcctccggtacctgcagccgccaccacttttcgaaggtaaatacctcgcggtgg
	agggtgcgcgggtgcggttgtgacgtcagggctgcccgagccctcgccggctcagcgggcggccacgcccccc gggctc
FOXO3-CDS	atggcagaggcaccggcttccccggccccgctctctccgctcgaagtggagctggacccggagttcgagccccagagccgtccgcgatcctg
	tacgtggcccctgcaaaggccggagctccaagcgagccctgccaagccctcgggggagacggccgccgactccatgatccccgaggagga
	ggacgatgaagacgacgaggacggcgggggacgggccggctcggccatggcgatcggcggcggcggcgggagcggcacgctgggctc
	cgggctgctccttgaggactcggcccgggtgctggcacccggagggcaagaccccgggtctgggccagccaccgcggcgggcgggctga
	gcgggggtacacaggcgctgctgcagcctcagcaaccgctgccaccgccgcagccgggggcggctgggggctccgggcagccgaggaa
	atgttcgtcgcggcggaacgcctggggaaacctgtcctacgcggacctgatcacccgcgccatcgagagctccccggacaaacggctcactc
	tgtcccagatctacgagtggatggtgcgttgcgtgccctacttcaaggataagggcgacagcaacagctctgccggctggaagaactccatccg
	gcacaacctgtcactgcatagtcgattcatgcgggtccagaatgagggaactggcaagagctcttggtggatcatcaaccctgatgggggaa
	gagcggaaaagcccccggcggcgggctgtctccatggacaatagcaacaagtataccaagagccgtggccgcgcagccaagaagaagg
	cagccctgcagacagcccccgaatcagctgacgacagtccctcccagctctccaagtggcctggcagccccacgtcacgcagcagtgatgag

ctggatgcgtggacggacttccgttcacgcaccaattctaacgccagcacagtcagtggccgcctgtcgcccatcatggcaagcacagagttgg

atgaagtccaggacgatgatgcgcctctctcgcccatgctctacagcagctcagccagcctgtcaccttcagtaagcaagccgtgcacggtgga

actgccacggctgactgatatggcaggcaccatgaatctgaatgatgggctgactgaaaacctcatggacgacctgctggataacatcacgctc

ccgccatcccagccatcgcccactgggggactcatgcagcggagctctagcttcccgtataccaccaagggctcgggcctgggctccccaac

cagctcctttaacagcacggtgttcggaccttcatctctgaactccctacgccagtctcccatgcagaccatccaagagaacaagccagctacctt

ctcttccatgtcacactatggtaaccagacactccaggacctgctcacttcggactcacttagccacagcgatgtcatgatgacacagtcggaccc

cttgatgtctcaggccagcaccgctgtgtctgcccagaattcccgccggaacgtgatgcttcgcaatgatccgatgatgtcctttgctgcccagcc

taaccagggaagtttggtcaatcagaacttgctccaccaccagcaccaaacccagggcgctcttggtggcagccgtgccttgtcgaattctgtca

gcaacatgggcttgagtgagtccagcagccttgggtcagccaaacaccagcagcagtctcctgtcagccagtctatgcaaaccctctcggactc

tctctcaggctcctccttgtactcaactagtgcaaacctgcccgtcatgggccatgagaagttccccagcgacttggacctggacatgttcaatgg

gagcttggaatgtgacatggagtccattatccgtagtgaactcatggatgctgatgggttggattttaactttgattccctcatctccacacagaatgt

tgttggtttgaacgtggggaacttcactggtgctaagcaggcctcatctcagagctgggtgccaggctg

Supplementary Table 6. Clinical characteristics of lung adenocarcinoma patients with MPE and different H3K18la levels.

Characteristics	H3K18la low (N=11)	H3K18la high (N=12)	P value
Age	59.91±10.07	63±8.312	0.4296
	(53, 66)	(56.25, 71.75)	
Sex			0.5518
Male	6 (54.55%)	8 (66.67%)	
Female	5 (45.45%)	4 (33.33%)	
Smoking			0.2138
Yes	3/11 (27.27%)	7 (58.33%)	
No	8 (72.73%)	5 (41.67%)	
Blood CEA (μg/ml)	48.51±66.39	126.4±220	0.2727
	(6.5, 58.3)	(19.63, 81.9)	
MPE CEA	359.4±454.3	855.2±609.3	0.0395*

(μg/ml)	(34.5, 1013)	(246.4, 1500)	
MPE LDH (U/L)	336.1±204.1	820.8±698.9	0.0382*
	(158, 559)	(383, 1023)	
